# Direct population of triplet excited states through singlet–triplet transition for visible-light excitable organic afterglow†
†Electronic supplementary information (ESI) available: Synthesis, characterization, experimental information and additional figures. CCDC 1573611, 1573612 and 1573614. For ESI and crystallographic data in CIF or other electronic format see DOI: 10.1039/c8sc05198d


**DOI:** 10.1039/c8sc05198d

**Published:** 2019-04-09

**Authors:** Jie Yuan, Runfeng Chen, Xingxing Tang, Ye Tao, Shen Xu, Lu Jin, Cailin Chen, Xinhui Zhou, Chao Zheng, Wei Huang

**Affiliations:** a Key Laboratory for Organic Electronics and Information Displays , Jiangsu Key Laboratory for Biosensors , Institute of Advanced Materials (IAM) , Jiangsu National Synergetic Innovation Center for Advanced Materials , Nanjing University of Posts & Telecommunications , 9 Wenyuan Road , Nanjing 210023 , China . Email: iamrfchen@njupt.edu.cn ; Email: wei-huang@njupt.edu.cn

## Abstract

Invoking efficient afterglow in metal-free organic molecules represents an important material advancement. However, organic afterglow suffers from low intensity and efficiency and generally needs to be excited by UV light owing to its spin-forbidden phosphorescent nature that essentially requires facile intersystem crossing (ISC). Here, we propose a strategy to bypass the traditional ISC through facilitating singlet–triplet transition to directly populate triplet excited states from the ground state by combining synergetic effects of both heavy/hetero-atom incorporation and aromatic aggregation. Verified by systematic experimental and computational investigations, this unique singlet-to-triplet absorption results in a much improved organic afterglow quantum efficiency up to 9.5% with a prolonged lifetime of 0.25 s under visible-light irradiation. Fundamentally, this work illustrates for the first time the great potential of the direct population method to red-shift the excitation wavelength and improve the afterglow efficiency, offering important clues for the development of triplet-state involved organic optoelectronic technologies.

## Abstract 2

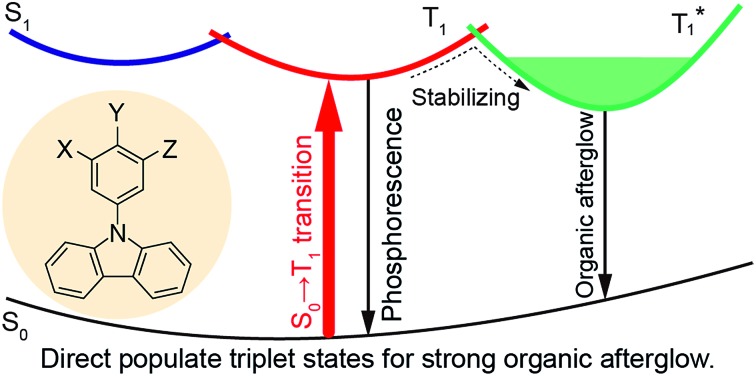
Direct population of triplet states *via* singlet-to-triplet absorption red-shifts the excitation wavelength and improves the organic afterglow efficiency under ambient conditions.

## Introduction

**Fig. 1:**
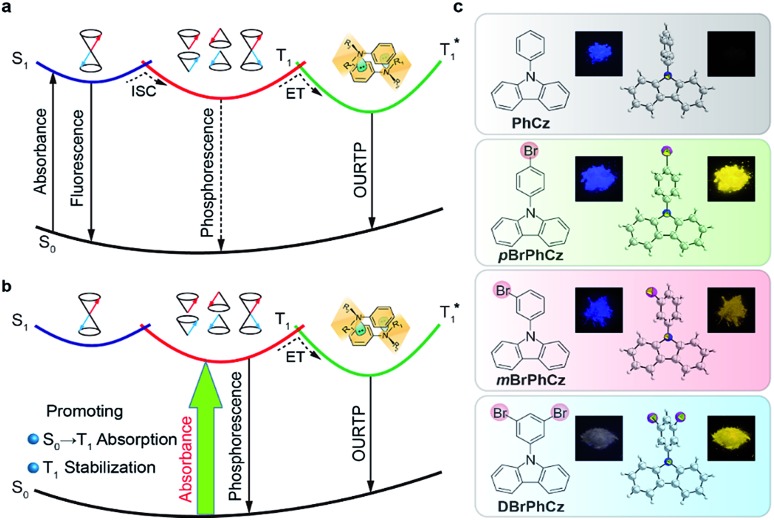
(a) Traditional mechanism for OURTP. (b) Proposed direct population of T_1_ through singlet-to-triplet (S_0_ → T_1_) absorption. (c) The S_0_ → T_1_ absorption enhanced OURTP molecules. Insets show the single crystal structures, PL photographs (left) upon 365 nm excitation, and afterglow photographs (right).

The triplet-state properties of metal-free organic materials have recently attracted broad attention because of their great potential for the development of low-cost and new featured luminescent materials, which may revolutionize current optical and electronic applications.^1^,^2^ Among various triplet-state involved optoelectronic phenomena, organic afterglow with an emission lifetime of over 100 milliseconds (ms) at room temperature is a unique example.^3^–^6^ Tremendous efforts have been devoted to revealing the mechanism of this extraordinary organic luminescence and various molecular design strategies have been proposed for the development of high-performance organic afterglow materials.^7^ Typically, afterglow appears when the photoexcited organic singlet excitons at the lowest singlet excited state (S_1_) transform effectively into triplet excitons *via* intersystem crossing (ISC), followed by exciton trapping (ET) in H-aggregation and radiative relaxation of the trapped triplet excitons to the ground state (S_0_) of organic small molecules (Fig. 1a).^3^ And, it has been widely acknowledged that organic afterglow is a kind of phosphorescence at room temperature due to the radiative decay of triplet excited states (T_*n*_) to induce organic ultralong room-temperature phosphorescence (OURTP) for afterglow emission, and efficient S_1_ → T_*n*_ ISC is essentially needed to transform photoexcited single excitons into triplet ones.^8^,^9^ Nevertheless, OURTP is essentially different from the thermally activated delayed fluorescence, which is fluorescence due to the radiative decay of S_1_, although the delayed part generated by facile reverse ISC from the lowest triplet excited state (T_1_) shows a lifetime up to several microseconds.^10^,^11^

To facilitate the ISC (S_1_ → T_*n*_) process for efficient spin-flipping of the excitons, tentative manipulation of the highly active singlet and triplet excited states to fulfil the basic requirements of both the energy gap law and El-Sayed rule is mandatory.^12^,^13^ The energy gap law requires matched energy levels of S_1_ and T_*n*_ with small singlet–triplet splitting energy (Δ*E*_ST_) to promote ISC, while the El-Sayed rule suggests that the ^1^(π, π*) → ^3^(n, π*) transition is faster than that of ^1^(π, π*) → ^3^(π, π*) and ^1^(n, π*) → ^3^(π, π*) is more facile than ^1^(n, π*) → ^3^(n, π*).^5^,^14^ Therefore, it is not an easy task to promote ISC in purely organic optoelectronic materials with a large Δ*E*_ST_ between S_1_ and T_1_ for significantly red-shifted organic afterglow; and, only a limited number of organic afterglow small molecules have been reported (Table S1†).^7^,^8^ Instead of attempting to promote the traditional S_1_ → T_*n*_ ISC, we decide to explore alternative methods to populate the triplet excited states of organic optoelectronic materials for organic afterglow (Fig. 1b). Considering that efficient room-temperature phosphorescence due to the spin-forbidden T_1_ → S_0_ transition has been widely recognized,^15^–^17^ we suppose that the reverse process of S_0_ → T_1_ transition may also be applicable to populate T_1_ without the participation of the S_1_ → T_*n*_ ISC, although its absorption cross section would be significantly lower than the spin-allowed excitation *via* S_0_ → S_1_ transition. Comprehensibly, the direct population of T_1_ through S_0_ → T_1_ transition, which bypasses the traditional ISC (S_1_ → T_*n*_) process, would red-shift the excitation wavelength to the visible range and improve the phosphorescence quantum efficiency (QE) by excluding the involvement of high-lying excited states and the unwanted relaxation processes.^18^ These advantages are even more important for organic afterglow, which is usually weak (QE < 5%) and needs to be excited with UV light (Scheme S1 and Table S1†) that is harmful to living beings. Nevertheless, the direct spin-forbidden excitation *via* S_0_ → T_1_ absorption is extremely weak in most organic materials, except for some metal complexes^18^–^20^ and aromatic molecules.^21^–^24^

Here, we succeed in designing a series of organic afterglow molecules capable of efficient direct S_0_ → T_1_ absorption by introducing heavy atoms of bromine into a heteroatom-containing molecule of 9-phenyl-9*H*-carbazole (**PhCz**). With the combined and synergetic effects of the heteroatom incorporation and internal and external heavy-atom interactions, enhanced S_0_ → T_1_ transition was successfully stimulated in these Br-substituted aromatic molecules. Excitingly, this S_1_ → T_*n*_ ISC-free strategy improves afterglow properties significantly with a prolonged emission lifetime to 0.25 s and a high QE up to 9.5% under visible light excitation (400 nm).^25^ The visible light-excitable organic afterglow with high QE is highly attractive for time-resolved imaging technologies, especially in bio-related applications. These findings illustrate the great feasibility and advantages of S_0_ → T_1_ absorption in metal-free organic materials to directly populate triplet states in stimulating long-lived and high-efficiency organic afterglow, providing fundamental understanding and important guidelines for the investigations and applications of triplet-state involved organic optoelectronic materials.

## Results and discussion

### Molecular design and characterization

To efficiently promote the S_0_ → T_1_ absorption, both heteroatom and heavy atom effects should be involved and combined synergistically in optoelectronically active π-conjugated systems with efficient internal interactions in single molecular states and external effects in crystals. Specifically, based on a weak organic afterglow molecule of **PhCz** with significant heteroatom effects of N, various numbers of heavy atoms of Br were introduced as substituents at different sites of benzene in **PhCz** to design various heavy-atom incorporated organic aromatic afterglow molecules of ***p*BrPhCz**, ***m*BrPhCz** and **DBrPhCz**, respectively (Fig. 1c). Their synthesis is convenient and efficient using a one-step Ullmann reaction in high yields (80–64%).^26^ Detailed synthetic procedures, molecular structure characterization, single crystal X-ray analysis, and thermal stability and electrochemical activity tests are presented in the ESI (Fig. S1–S10, Tables S2 and S3†).

### Photophysical property investigation

**Fig. 2:**
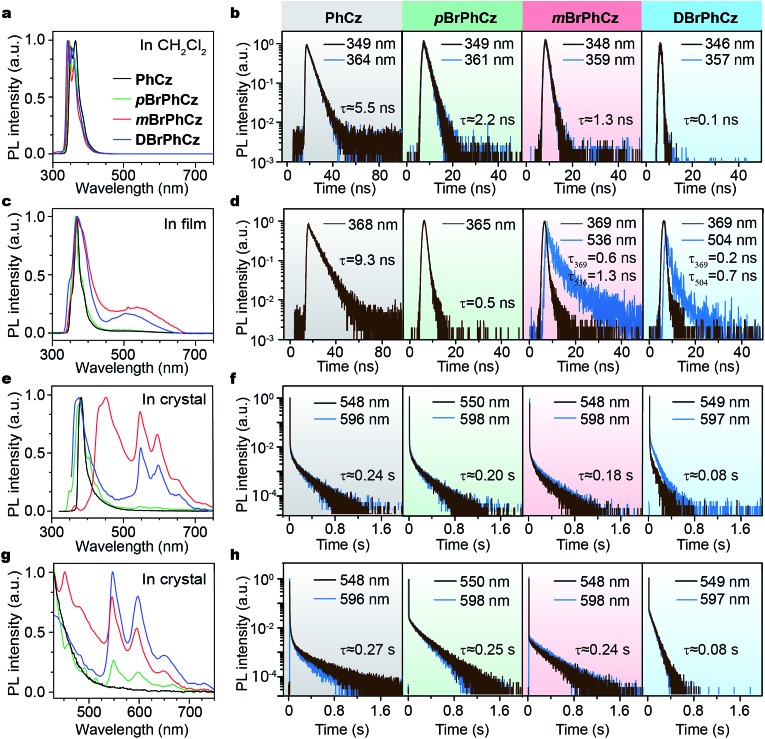
(a–h) Room-temperature steady-state emission spectra and lifetime decay profiles in dilute CH_2_Cl_2_ solution (a and b), a thin film (c and d), and a crystal (e and f) excited at 295 nm, as well as in a crystal excited at 400 nm (g and h).

The Br-substituted **PhCz** compounds have very similar UV-vis absorption spectra to **PhCz** in dilute solutions, thin films, and crystals (Fig. S11 and Table S4†). The characteristic fluorescence emission bands of the carbazole moiety in these compounds were observed in their PL spectra in both solution (Fig. 2a and b) and films (Fig. 2c and d). Interestingly, when these molecules are in crystals, extraordinary OURTP for organic afterglow emission appears; accompanied by the fluorescence bands, the OURTP emission peaks around 550 and 597 nm can even be observed in the stead-state photoluminescence (PL) spectra (Fig. S12 and S13†). More interestingly, the lifetime of the emission reaches 0.20 s in ***p*BrPhCz** crystals under the UV-light (295 nm) excitation (Fig. 2e and f), but when these crystals are excited at 400 nm, stronger afterglow emission with a longer lifetime in the Br-substituted compounds is observed, exhibiting an OURTP lifetime up to 0.25 s and a QE of 9.5%. To our knowledge, this is the highest efficiency upon visible-light excitation and also among the best results of UV-light excited organic afterglow ever reported.^7^,^25^

**Fig. 3:**
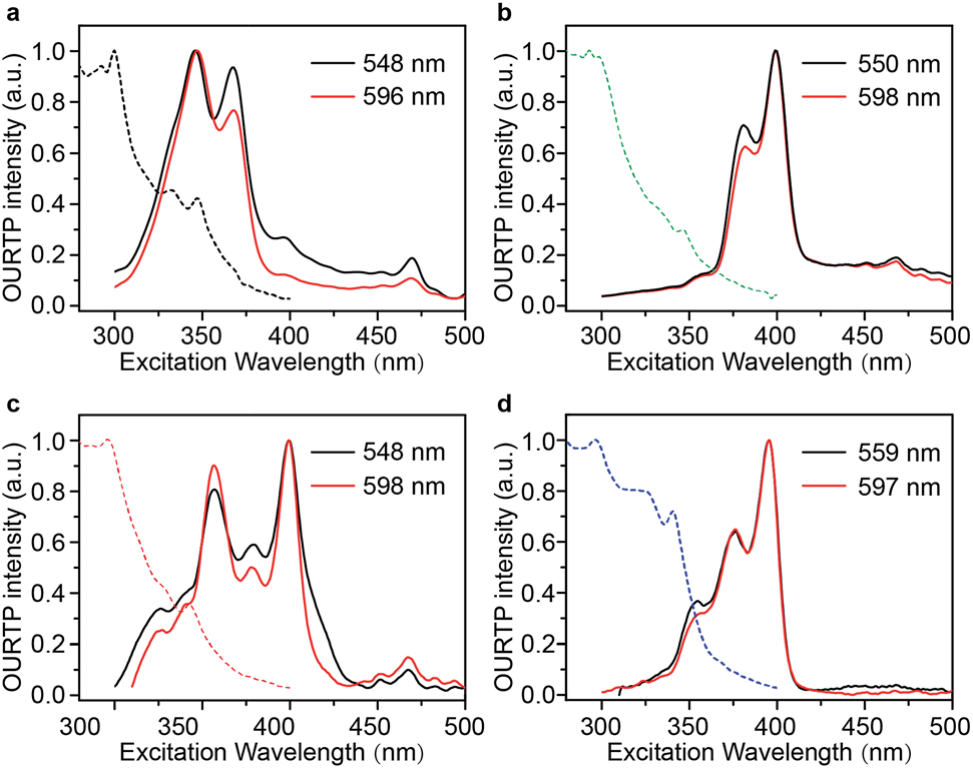
UV-vis absorption (dotted line) and excitation (solid line) spectra for OURTP peaks of **PhCz** (a), ***p*BrPhCz** (b), ***m*BrPhCz** (c) and **DBrPhCz** (d) crystals at room temperature.

**Fig. 4:**
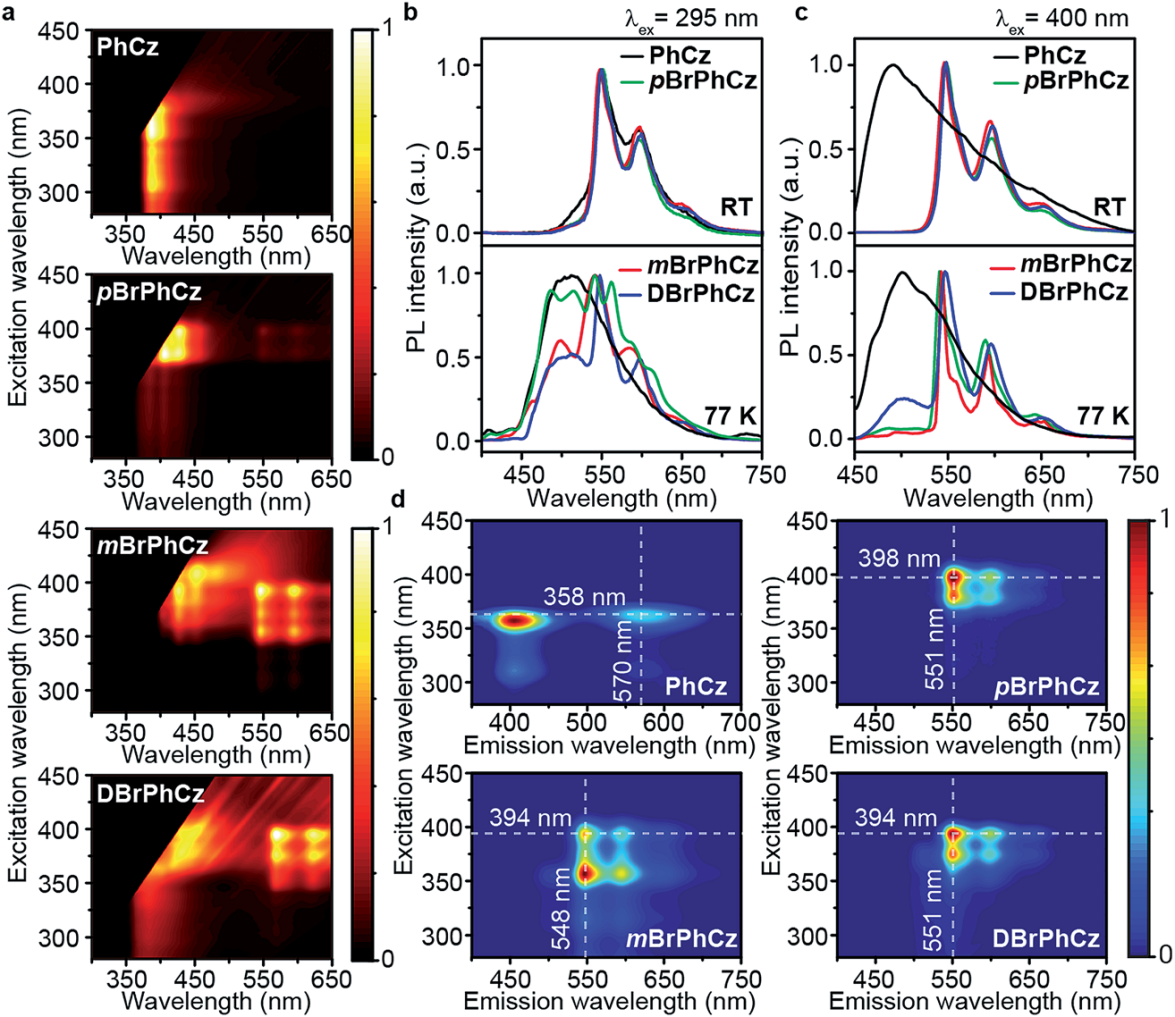
UV and visible-light excited organic afterglow of **PhCz**, ***p*BrPhCz**, ***m*BrPhCz** and **DBrPhCz** crystals. (a) Steady-state excitation–emission mapping at room temperature. (b and c) OURTP spectra under 295 (b) and 400 nm (c) excitation with a delay time of 40 ms at room temperature (RT) and 200 ms at 77 K. (d) Excitation-phosphorescence mapping with 5 ms delay at room temperature.

Excitation spectra of the OURTP peaks (Fig. 3) were investigated to explore the extraordinarily efficient visible-light excited afterglow emission with a prolonged lifetime up to 33% and an enhanced QE (6.3 fold) compared to the 295 nm excited OURTP emission.^27^ The afterglow emission of **PhCz** crystals can be hardly excited at 400 nm with a weak excitation shoulder, while that of the brominated compounds is effectively photoexcited with visible light at room temperature and the wavelength can range from 350 to 470 nm in ***m*BrPhCz** crystals at 77 K, although the absorbance in the visible range is relatively very low (Fig. 3 and S14†). From the steady-state excitation–emission mapping (Fig. 4a), afterglow emission around 550 and 597 nm is very weak in **PhCz** crystals, while after the Br-substitution, the afterglow enhances significantly and can be excited more facilely by the visible light; this is especially obvious in ***m*BrPhCz** crystals, showing significantly strengthened afterglow peaks when the excitation wavelength is longer than 350 nm at room temperature.

Time-resolved spectroscopy techniques were further applied to study the unexpected visible-light excited organic afterglow. With a delay time of more than 40 ms, short-lived fluorescence in ns and phosphorescence in ms can be effectively eliminated, leaving only long-lived OURTP emission.^28^ Therefore, identical yellow afterglow with three resolved emission bands at 549, 597, and 650 nm of these brominated molecules in crystals was observed, when excited with either 295 or 400 nm light at room temperature (Fig. 4b and c). At a low temperature of 77 K, their OURTP spectra vary significantly after 295 nm excitation, showing newly emerged OURTP peaks with a shorter emitting wavelength; this blue-shifted OURTP band is suppressed after 400 nm excitation, suggesting that different photophysical processes are involved when excited with UV-light (295 nm) and visible light (400 nm). Furthermore, time-resolved excitation–emission mapping with a 5 ms delay also shows strong and dominated afterglow emission in crystals of Br-substituted **PhCz** compounds, when excited with visible light at room temperature (Fig. 4d). These observations confirm the significantly enhanced afterglow emission after visible light excitation, when heavy atoms of Br are introduced into **PhCz**.

How can the afterglow emission be effectively excited by the 400 nm visible light, which is even longer in wavelength than their fluorescence peaks (345–365 nm)? To understand this phenomenon, phosphorescence spectra of these compounds were investigated in dilute solution at 77 K with a delay time of 10 ms (Fig. S15†).^29^ Under the UV-light excitation at 295 nm, very similar phosphorescence spectra showing typical triplet features of carbazole with similar T_1_ energies (∼3.03 eV) for these compounds were observed. With the 400 nm visible-light excitation, almost identical phosphorescence spectra were obtained, suggesting that T_1_ can be populated by 400 nm photoexcitation even at the single molecular state in dilute solution, since the 400 nm excitation and 408 nm phosphorescence are very close in energy. Moreover, the OURTP intensity of ***p*BrPhCz** crystals at room temperature (Fig. S16†) increases linearly to the 400 nm excitation strength with constant QEs (Fig. S17†), ruling out the possibility of multiphoton processes in the photoexcitation for OURTP. Therefore, we propose that the T_1_ could be directly populated under visible-light excitation through S_0_ → T_1_ absorption.

The OURTP emission of these heavy-atom substituted and heteroatom incorporated molecules can be excited with either 295 or 400 nm light with varied intensities and irradiation times and is very stable in both strength and lifetime in different atmospheres of nitrogen, air and oxygen (Fig. S18–S21†). Therefore, two excitation ways are suspected to work here to populate T_1_ for both phosphorescence and OURTP emission (Table 1). The first way is traditional, through spin-allowed photoabsorption to populate S_1_, followed by S_1_ → T_*n*_ ISC to populate T_1_ for phosphorescence and energy trapping (ET) to form stabilized 
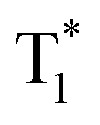
 in aggregated structures for OURTP.^30^ The other possible way is directly through S_0_ → T_1_ absorption, followed by ET to produce 
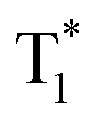
 for OURTP.

**Table 1:** Luminescence properties under UV (295 nm) and visible light (400 nm) excitation at room temperature

Compd.	Fluorescence (*λ*_ex_ = 295 nm)						OURTP^*c*^ (*λ*_ex_ = 295 nm)			OURTP^*c*^ (*λ*_ex_ = 400 nm)	
	*λ* ^*a*^ (nm)	*τ* ^*a*^ (ns)	*λ* ^*b*^ (nm)	*τ* ^*b*^ (ns)	*λ* ^*c*^ (nm)	*τ* ^*c*^ (ns)	*λ* (nm)	*τ* (s)	*η*_p_ (%)	*τ* (s)	*η*_p_ (%)
**PhCz**	349/364	5.5	368	9.3	380	11.2	548/596	0.24/0.23	1.5	0.27/0.26	1.8
***p*BrPhCz**	349/361	2.2	365	0.5	385	3.9	550/598	0.20/0.20	1.5	0.25/0.23	9.5
***m*BrPhCz**	348/359	1.3	369	0.6	432	6.6	548/598	0.18/0.17	3.3	0.24/0.23	6.6
***D*BrPhCz**	346/357	0.1	369	0.2	375	8.5	549/597	0.08/0.07	1.6	0.08/0.08	8.2

^*a*^In CH_2_Cl_2_.

^*b*^In a thin film.

^*c*^In a crystal.

**Fig. 5:**
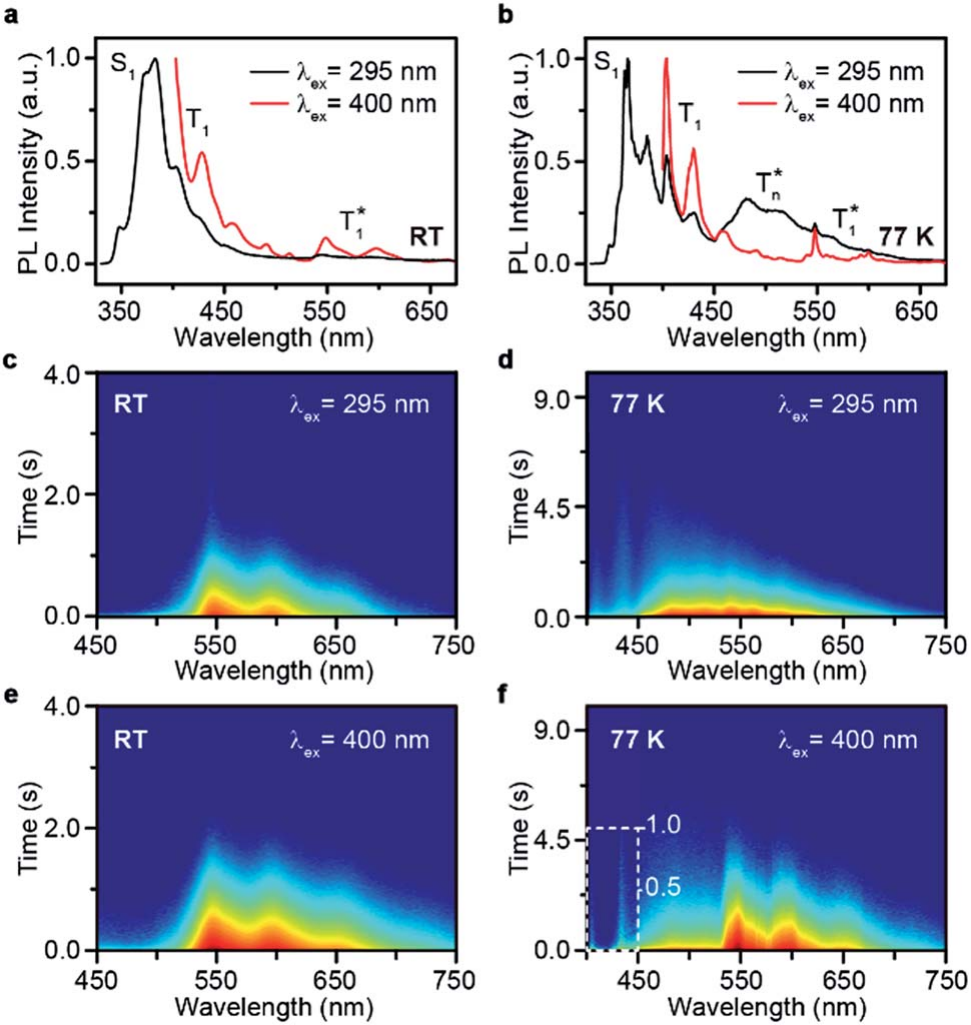
(a–f) Steady-state PL spectra (a and b) and transient PL decay images (c–f) of ***p*BrPhCz** crystals excited with 295 (a, c, and e) and 400 nm (b, d, and f) light at room temperature (RT) and 77 K.

Take ***p*BrPhCz** crystals as a typical example. At room temperature, only weak OURTP peaks with strong fluorescence can be observed in the steady-state PL spectrum under 295 nm excitation; when excited at 400 nm, both strong phosphorescence peaked at 430 nm and OURTP at 550 and 598 nm appear (Fig. 5a). It should be noted that these phosphorescence peaks in the crystals are very close to those in dilute solution (Fig. S15†) and their phosphorescence nature was further confirmed by their long lifetime (26 ms, Fig. S22†). When the temperature drops to 77 K, the non-radiative relaxation processes of the excited states are significantly suppressed, leading to enhanced, refined and prolonged fluorescence, phosphorescence and OURTP emissions (Fig. 5b). However, a new broad emission band emerges around 482 nm under 295 nm excitation at 77 K. Transient PL decay images show that this emission band has a long lifetime (0.41 s), which confirms its phosphorescent nature with significantly different afterglow emission profiles at 77 K in comparison to that at room temperature (Fig. 5c and d). Since this long-lived broad emission appears only at low temperature under 295 nm excitation but is absent under 400 nm excitation even at 80 K (Fig. S23†), we ascribe it to the radiative decay of the high-lying triplet excitons stabilized in aggregated structures (
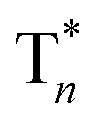
);^31^ the low temperature blocks, to some extent, the internal conversion (IC) between triplet excited states. Under 400 nm excitation, almost identical OURTP emission profiles were observed at either room temperature or 77 K to those under 295 nm excitation (Fig. 5e and f). Again, the phosphorescent nature of the 548 and 600 nm emission bands becomes more evident at 77 K. Therefore, it can be inferred that both 295 and 400 nm light can excite the molecule to triplet excited states for the same phosphorescence and OURTP spectra but in different ways with different quantum efficiencies and lifetimes at either room temperature or 77 K.

### Proposed mechanism in direct population of T_1_

**Fig. 6:**
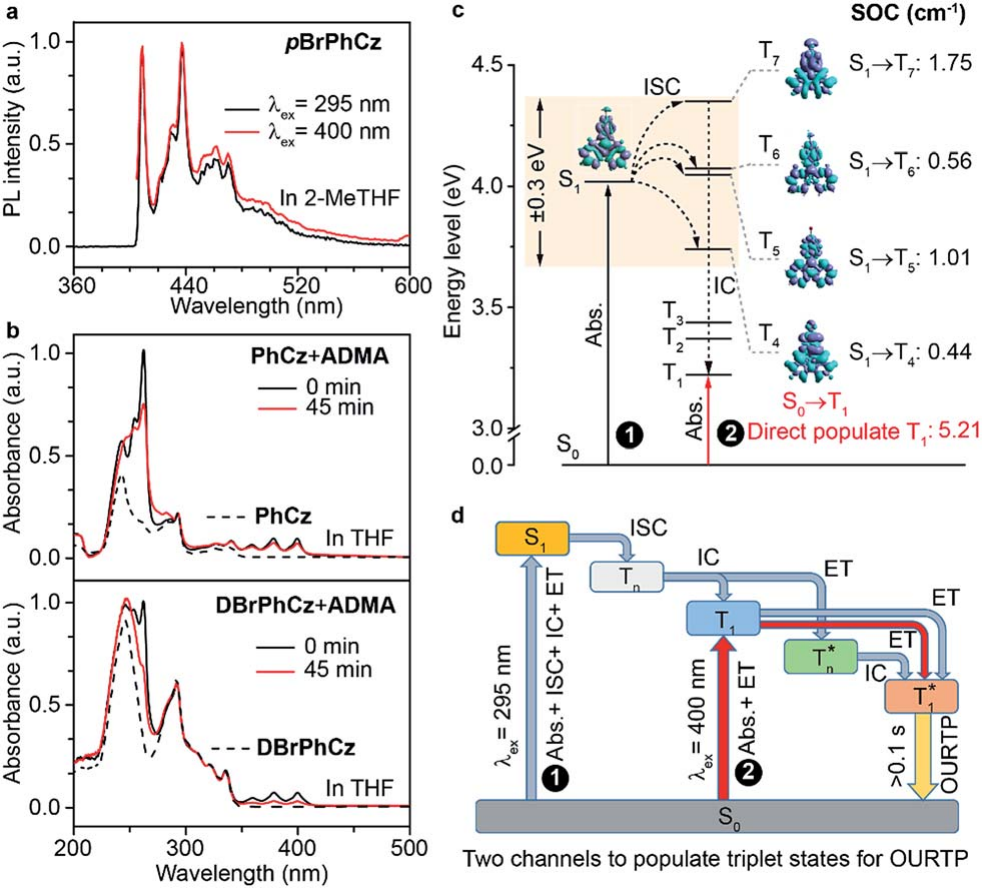
(a) Phosphorescence spectra of ***p*BrPhCz** in 2-methyl tetrahydrofuran (2-Me THF) at 77 K with a delay time of 10 ms after the 295 (black line) and 400 nm (red line) excitation. (b) absorption spectra of **PhCz** (20 μM, upper, dashed line), **DBrPhCz** (20 μM, below, dashed line) and their mixtures with ADMA (50 μM, solid line) in THF after the excitation at 400 nm for 0 (black line) and 45 min (red line). (c) TD-DFT-calculated energy levels, EDD, and SOC values of the singlet and triplet excited states of ***p*BrPhCz**. (d) OURTP through two pathways.

Besides the 400 nm excitable phosphorescence (Fig. 6a) to verify the direct population of T_1_, photodegradation of anthracene-9,10-diyl-bis-methylmalonate (ADMA) in the presence of OURTP molecules and oxygen was also performed (Fig. S24, Section 6.1†).^32^ The characteristic absorption peaks (359, 378 and 399 nm) of ADMA in **DBrPhCz** solution gradually decrease in intensity with prolonged irradiation time, directly indicating the existence of triplet excited states upon 400 nm photoexcitation; and, the more rapidly decreased ADMA absorption peaks in **DBrPhCz** solution indicate more efficient direct formation of triplet excited states with the aid of multiple heavy atom effects of **DBrPhCz** (Fig. 6b). Control experiments were also performed to check the unique S_0_ → T_1_ absorption in these heavy atom and heteroatom incorporated molecules; the previously reported afterglow molecule of **DNCzP**, which has a similar phosphorescence spectrum to ***p*BrPhCz**, cannot be excited by the visible light for either phosphorescence or afterglow emission (Fig. S25†).^3^

To theoretically understand the direct S_0_ → T_1_ absorption, we performed first-principles time-dependent density functional theory (TD-DFT) investigations on these molecules (Fig. 6c).^33^ The calculated S_1_ energy levels are considerably close (<0.3 eV) to several triplet excited states (T_*n*_), supporting facile ISC channels for S_1_ → T_*n*_ transitions according to the energy gap law. Moreover, high spin-orbital coupling (SOC) values with similar electron density difference (EDD) isosurfaces further indicate the efficient ISC of these transition channels according to the El-Sayed rule (Fig. S26†). Notably, these SOC values are significantly larger than those of heteroatom-free or heavy atom-free organic molecules (<0.1 cm^–1^).^34^ Thus, the 295 nm excited S_1_ can be transformed into T_*n*_*via* these ISC channels, and the T_*n*_ can be relaxed to T_1_ for phosphorescence and OURTP in the traditional path to populate triplet states. It should also be noticed that wide energy gaps (up to 0.5 eV) between the different T_*n*_ energy levels may hinder the IC of T_*n*_ to reach T_1_, especially at low temperatures; the radiative decay of 
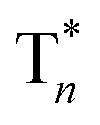
 in crystals could be responsible for the new emission bands (∼480 nm) upon UV-light excitation at 77 K (Fig. 5b).^31^ More importantly, very high SOC values of S_0_ → T_1_ transition, which are even much higher than those of S_1_ → T_*n*_ transitions, were revealed in these heteroatom and heavy atom modified molecules, offering computational evidence for the direct S_0_ → T_1_ absorption. It should be noted that the SOC calculation is based on isolated single molecular states, which may overlook the intermolecular heavy atom effects in promoting SOC. Indeed, apparent weak interactions between Br and the aromatic moiety of its adjacent molecule can be figured out through non-bonding covalent interaction (NCI) analysis (Fig. S27†).^35^,^36^ Therefore, even more efficient S_0_ → T_1_ transition can be expected in the condensed solid states of these brominated molecules.

On the basis of these experimental and theoretical evidence, a two-way OURTP mechanism for visible-light excitable OURTP can be proposed (Fig. 6d). The first way is traditional, containing four steps of photoabsorption, S_1_ → T_*n*_ ISC, IC between T_*n*_, and triplet exciton trapping (ET). H-aggregation, which is important in trapping and stabilizing the triplet excited states for efficient OURTP,^3^ can be figured out clearly in these Br-substituted afterglow molecules by positive exciton splitting energy according to the molecular exciton theory (Fig. S28–S30 and Table S6†).^37^ The second way to populate T_1_ for afterglow is through direct S_0_ → T_1_ absorption, which bypasses the traditional ISC (S_1_ → T_*n*_) and IC processes. Since it is spin-forbidden with a large geometry difference between S_0_ and T_1_, the S_0_ → T_1_ absorption is weak and slow, and needs more time to populate T_1_ for the steady emission of OURTP according to the Franck–Condon principle. Nevertheless, this way for OURTP involves only two excited states of T_1_ and 
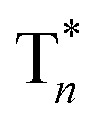
 , while the radiative and non-radiative decays of other excited states needed in the first way are completely avoided. Therefore, significantly improved OURPT quantum efficiencies up to 9.5% under 400 nm excitation were achieved by direct S_0_ → T_1_ absorption, showing a 6.3 fold enhancement in comparison with that traditionally achieved under UV-light excitation.

### Lifetime-resolved and color-encoded flexible pattern encryption

**Fig. 7:**
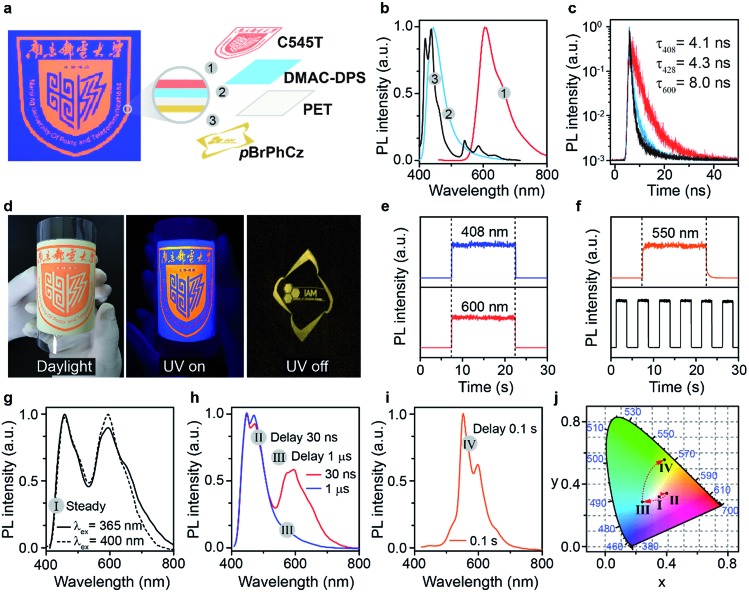
(a) Device structure of the flexible pattern encryption. (b and c) Steady state PL spectra (b) and fluorescence lifetime decay curves (c) of the red, blue, and yellow layers under 395 nm irradiation. (d) Photographs of the device under daylight (left) and 365 nm irradiation (middle), as well as after the removal of the excitation light (right). (e and f) Time-dependent intensity profiles of the 408 and 600 nm fluorescence (e) and OURTP emission (f) at 550 nm. (g–j) PL spectra (g–i) and color coordinates (j) of the device at a steady state (I, g) and after different delay times of 30 ns (II, h), 1.0 μs (III, h), and 0.1 s (IV, i) under 400 nm excitation.

In light of the extraordinary high-efficiency visible-light excitable OURTP emission by direct S_0_ → T_1_ absorption, lifetime-resolved and color-encoded pattern encryption^16^,^38^ becomes possible using these heavy atom and heteroatom incorporated OURTP molecules and commercially available fluorescent compounds on flexible polyethylene terephthalate (PET) substrates (Fig. 7). The ‘NJUPT’ logo pattern was painted in red using the fluorescent dye of 10-(2-benzothiazolyl)-2,3,6,7-tetrahydro-1,1,7,7-tetramethyl-1*H*,5*H*,11*H*-(1)benzopyropyrano(6,7-8-I,*j*)quinolizin-11-one (C545T) on the surface of a blue emitting layer of bis-4-(9,9-dimethyl-9,10-dihydroacridine)phenylsulfone (DMAC-DPS) on one side of a PET substrate; on the other side of the substrate, an ‘IAM’ logo was painted using the OURTP molecule of ***p*BrPhCz** (Fig. S31†). The fluorescent and OURTP emitters show a similar steady state emission color so that the bottom pattern of the ‘IAM’ logo is well encrypted, showing only the reddish ‘NJUPT’ logo under a 365 nm UV lamp; once the excitation lamp was turned off, the ‘IAM’ logo in yellow afterglow emission can be clearly observed with the naked eye (Fig. 7a–d). This convenient time-resolved encryption technology containing short-lived blue (408 nm) and red (600 nm) emission channels and a long-lived yellow (550 nm) OURTP channel (Fig. 7e and f) also contains color-encoded pattern encryption,^28^,^39^ when the luminescence spectra evolve after the switching-off of the excitation (Fig. 7g–j).

## Conclusion

In summary, we succeed in developing a series of organic afterglow molecules capable of direct triplet excited state population upon photoexcitation through efficient singlet-to-triplet absorption by incorporating both heteroatoms and heavy atoms synergistically in aromatic organic crystals. The facilitated S_0_ → T_1_ transition, verified by combined experimental and theoretical investigations, brings about unique features of organic afterglow, including visible-light excitability, prolonged emission lifetime and significantly improved QE under ambient conditions, in contrast to traditional ISC-involved OURTP emission under UV-light irradiation. Moreover, based on the strong and efficient afterglow, a lifetime-resolved and color-encoded flexible pattern encryption device can be successfully fabricated. Importantly, the direct population of triplet excited states by S_0_ → T_1_ absorption represents a significant concept advancement in constructing triplet-state involved materials, breaking the traditional belief that S_1_ → T_*n*_ ISC is mandatory to populate T_1_ upon photoexcitation. These findings with new insights into the population of triplet excited states of metal-free organic optoelectronic molecules would promote significantly the development and applications of triplet-state involved organic materials.

## Conflicts of interest

The authors declare no conflict of interest.

## Supplementary Material

Supplementary information

Supplementary movie

Crystal structure data
