# Supplementary material for: Direct population of triplet excited states through singlet–triplet transition for visible-light excitable organic afterglow
Source: Chem Sci. 2019 Apr 9;10(19):5031–8. doi: 10.1039/c8sc05198d (PMC6530535; doi:10.1039/c8sc05198d)
Supplement: Supplementary file 1 [file SC-010-C8SC05198D-s001.pdf]

Electronic Supplementary Material (ESI) for Chemical Science.
This journal is © The Royal Society of Chemistry 2019

Electronic Supporting Information (ESI)

Direct Populating Triplet Excited States through Singlet-triplet Transition for Visible-Light Excitable Organic Afterglow

Jie Yuan, Runfeng Chen, Xingxing Tang, Ye Tao, Shen Xu, Lu Jin, Cailin Chen, Xinhui Zhou, Chao Zheng, Wei Huang**

Key Laboratory for Organic Electronics and Information Displays & Jiangsu Key Laboratory for Biosensors, Institute of Advanced Materials (IAM), Jiangsu National Synergistic Innovation Center for Advanced Materials (SICAM), Nanjing University of Posts & Telecommunications, 9 Wenyuan Road, Nanjing 210023, China

**Email: iamrfchen@njupt.edu.cn, wei-huang@njupt.edu.cn*

Contents

1. Synthesis and characterization 2
2. A brief survey of published organic afterglow molecules 6
3. Single crystal X-ray analysis 7
4. Thermophysical and electrochemical property measurements 9
5. Photophysical property investigations 10
   5.1 Steady-state photophysical properties 11
   5.2 Time-resolved photoluminescence properties 15
   5.3 Excitation strength and duration consideration 19
6. Mechanism study 22
   6.1 Probing the formation of triplet excited states 22
   6.2 Control experiment 23
   6.3 Theoretical calculations 24
   6.4 Photophysical modeling 27
7. Aggregation structure analysis 29
8. Flexible pattern encryption application 33

1. Synthesis and characterization

Chemicals and solvents purchased from Aldrich or Acros are of analytical grade and were used without further purification. Unless otherwise noted, reactions were carried out under a dry nitrogen atmosphere using standard Schlenk techniques. ^{1}H and ^{13}C-nuclear magnetic resonance (NMR) spectra were recorded on a Bruker Ultra Shield Plus 400 MHz instrument with $CDCl_3$ as the solvent and tetramethylsilane (TMS) as the internal standard. Chemical shifts (δ) are given in *ppm* in Hz. Splitting patterns were designed as follows: s (singlet), d (doublet) and m (multiplet). Mass spectra were obtained using a Shimadzu GCMS-QP2010 instrument. Elemental analyses were performed on an Elementar Vario MICRO elemental analyzer.

9-Phenyl-9H-carbazole (PhCz): To a 250 mL round bottom flask charged with a stir bar was added 9H-carbazole (3.34 g, 20.0 mmol), copper (0.56 g, 8.8 mmol), potassium carbonate (11.17 g, 80.8 mmol), bromobenzene (3.2 mL, 4.70 g, 30.0 mmol), and 50 mL dry nitrobenzene. The mixture under nitrogen protection was stirred and reacted for 24 hours at 180°C. After cooling to room temperature, the solvent of nitrobenzene was removed by vacuum distillation.[1] The resulting solid was dissolved in 120 mL dichloromethane (DCM) and washed with brine (60 mL). The mixture was then extracted with DCM for three times. The organic phase was collected and dried over $MgSO_4$. After removing the solvent under reduced pressure, the crude product was purified by column chromatograph (using petroleum ether: DCM = 10:1 as the eluent) and recrystallized from DCM/hexane for several times to obtain a colorless crystal. Yield: 4.20 g (86%). ^{1}H NMR (400 MHz, $CDCl_3$): δ= 8.21-8.19 (d, 2H), 7.66-7.60 (m, 4H), 7.52-7.43 (m, 5H), 7.34-7.33 (m, 2H). ^{13}C NMR (100 MHz, $CDCl_3$): δ= 140.95, 137.77, 129.92, 127.49, 127.20, 125.97, 123.41, 120.35, 119.95, 109.82. GC-MS (CH_2Cl_2): *m/z* calcd. for $C_{18}H_{13}N$ $[M+H]^+$: 243.1; found: 242.9. Anal. calcd. for C18H13N: C 88.86, H 5.39, N 5.76; found: C 88.64, H 5.42, N 5.39.

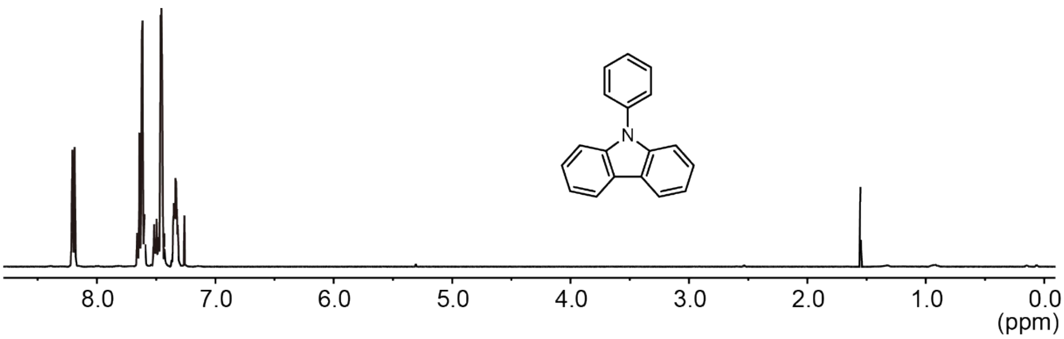

Figure S1. ^{1}H NMR spectrum of 9-phenyl-9H-carbazole (**PhCz**).

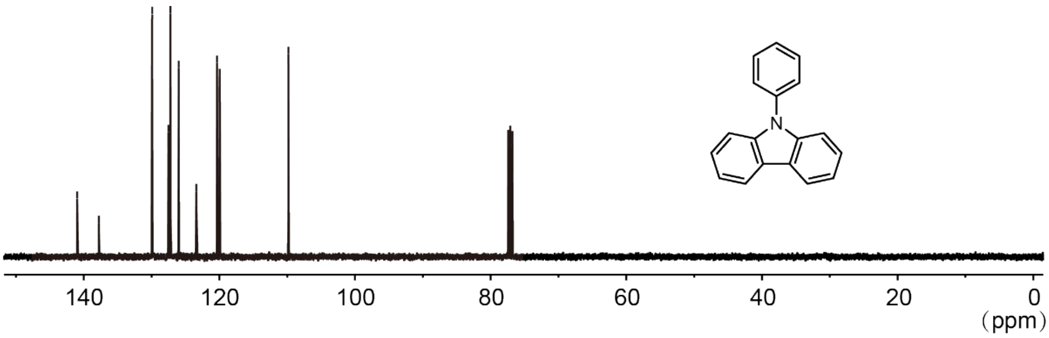

Figure S2. ^{13}C NMR spectrum of 9-phenyl-9H-carbazole (**PhCz**).

9-(4-Bromophenyl)-9H-carbazole (*p*BrPhCz): *p*BrPhCz was prepared in a same procedure to that of **PhCz**, but instead of using bromobenzene, 1,4-dibromobenzene (7.00 g, 30.0 mmol) was used as the starting material. Yield: 7.70 g of colorless crystal (80%). ^{1}H NMR (400 MHz, $CDCl_3$): δ= 8.16-8.14 (d, 2H), 7.75-7.73 (d, 2H), 7.47-7.37 (m, 6H), 7.33-7.29 (m, 2H). ^{13}C NMR (100 MHz, $CDCl_3$): δ= 140.61, 136.81, 133.13, 128.74, 126.11, 123.49, 120.91, 120.42, 120.23, 109.56. GC-MS (CH_2Cl_2): *m/z* calcd. for $C_{18}H_{12}NBr$ $[M+H]^+$: 322.0; Found: 322.9. Anal. calcd. for $C_{18}H_{12}NBr$: C 67.10, H 3.75, N 4.35; found: C 66.49, H 3.80, N, 3.96.

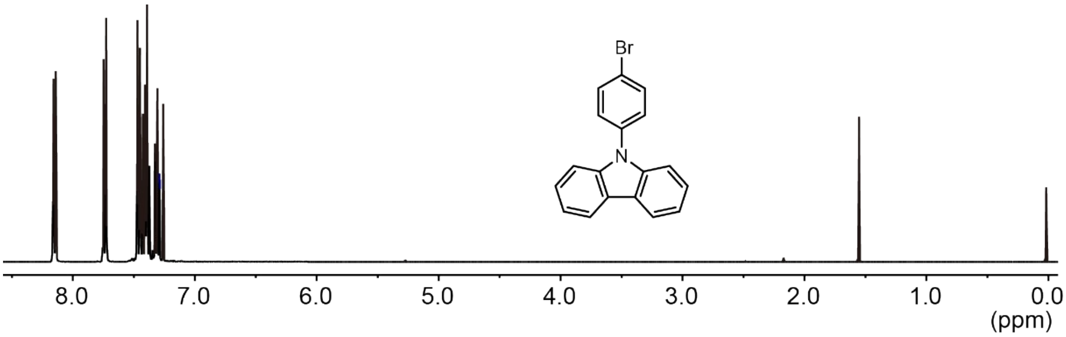

Figure S3. ^{1}H NMR spectrum of 9-(4-bromophenyl)-9H-carbazole (***p*BrPhCz**).

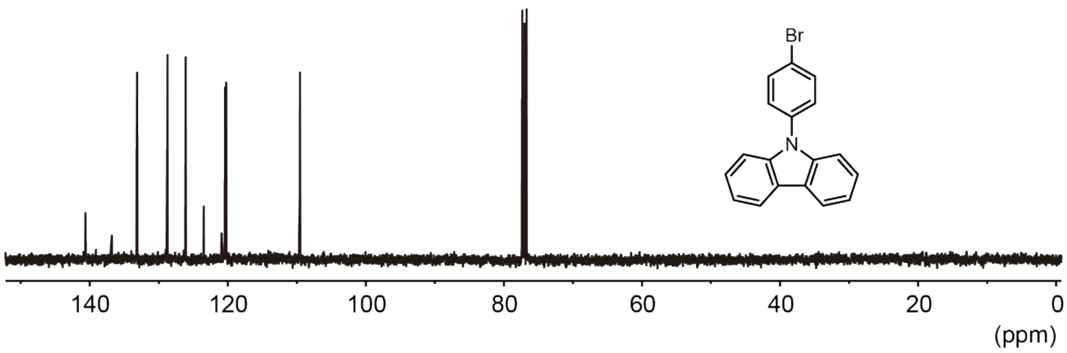

Figure S4. ^{13}C NMR spectrum of 9-(4-bromophenyl)-9H-carbazole (***p*BrPhCz**).

9-(3-Bromophenyl)-9H-carbazole (*m*BrPhCz): Similar to the preparation of **PhCz**, ***m*BrPhCz** was synthesized by using 4.8 mL *m*-dibromobenzene (7.05 g; 30.0 mmol). Yield: 4.60 g of colorless crystal (72%). ^{1}H NMR (400 MHz, $CDCl_3$): δ= 8.30-8.28 (d, 2H), 7.90 (s, 1H), 7.71-7.69 (d, 1H), 7.61-7.44 (m, 8H). ^{13}C NMR (100 MHz, $CDCl_3$): δ= 140.58, 139.15, 131.15, 130.55, 130.18, 126.16, 125.76, 123.56, 123.25, 120.43, 120.37, 109.65. GC-MS (CH_2Cl_2): *m/z* calcd. for $C_{18}H_{12}NBr$ $[M+H]^+$: 322.0; Found: 322.9. Anal. calcd. for $C_{18}H_{12}NBr$: C 67.10, H 3.75, N 4.35; found: C 66.94, H 3.87, N 3.98.

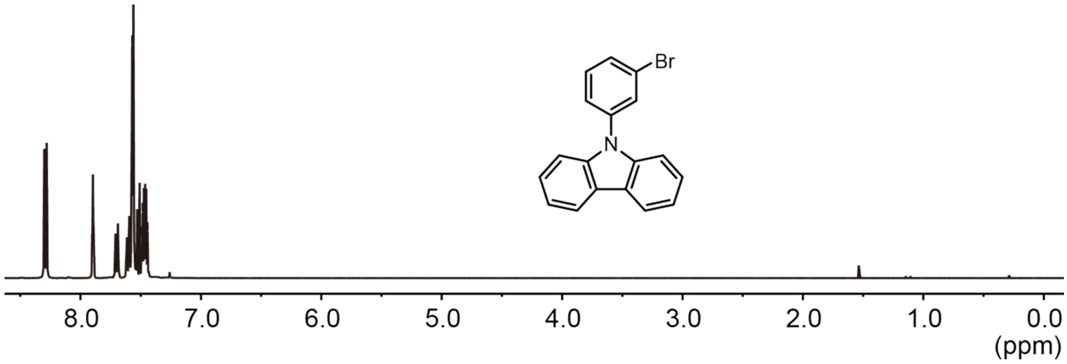

Figure S5. ^{1}H NMR spectrum of 9-(3-bromophenyl)-9H-carbazole (***m*BrPhCz**).

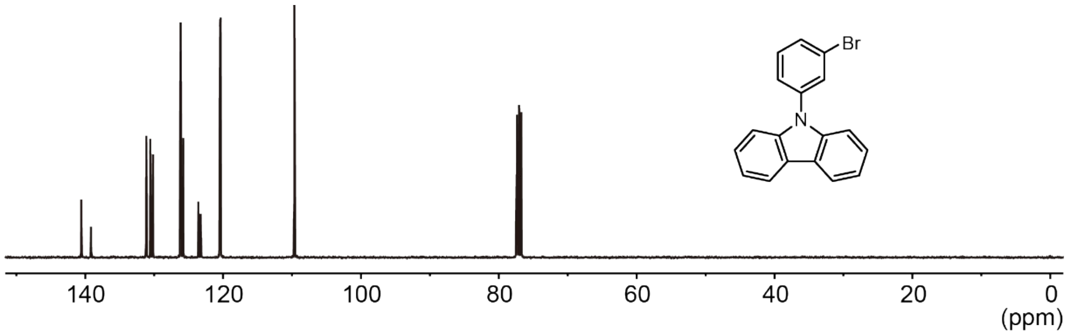

Figure S6. ^{13}C NMR spectrum of 9-(3-bromophenyl)-9H-carbazole (***m*BrPhCz**).

9-(3,5-Bibromophenyl)-9H-carbazole (DBrPhCz): Similarly, **DBrPhCz** was prepared from 1,3,5-tribromobenzene (11.97 g; 30 mmol). Yield: 5.10 g of colorless crystal (64%). ^{1}H NMR (400 MHz, $CDCl_3$): δ= 8.14-8.12 (d, 2H), 7.77 (t, 1H), 7.71 (d, 2H), 7.70-7.41 (m, 4H), 7.3-7.30 (m, 2H). ^{13}C NMR (100 MHz, $CDCl_3$): δ= 140.82, 140.23, 140.09, 132.99, 128.83, 126.34, 123.75, 120.77, 120.50, 109.52. GC-MS (CH_2Cl_2): *m/z* calcd. for $C_{18}H_{11}NBr_2$ $[M+H]^+$: 401.1; found: 400.6. Anal. calcd. for $C_{18}H_{11}NBr_2$: C 53.90, H 2.76, N 3.49; found: C 54.09, H 3.01, N 3.09.

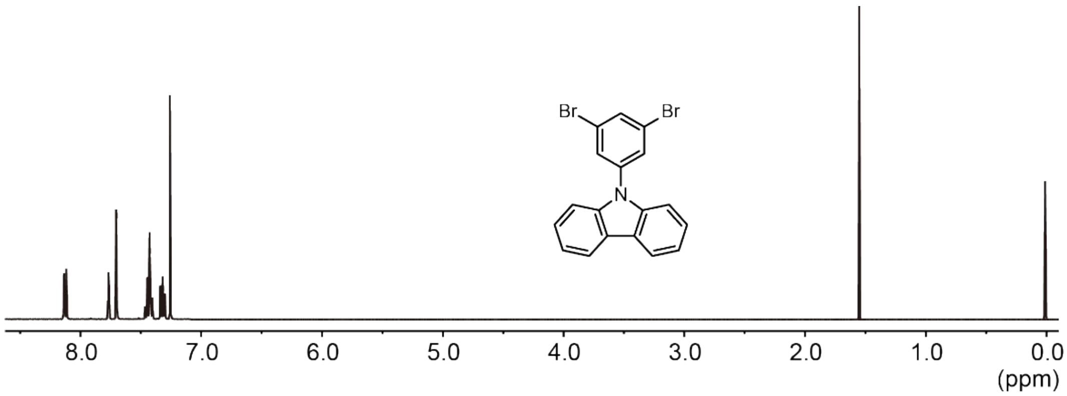

Figure S7. ^{1}H NMR spectrum of 9-(3,5-dibromophenyl)-9H-carbazole (**DBrPhCz**).

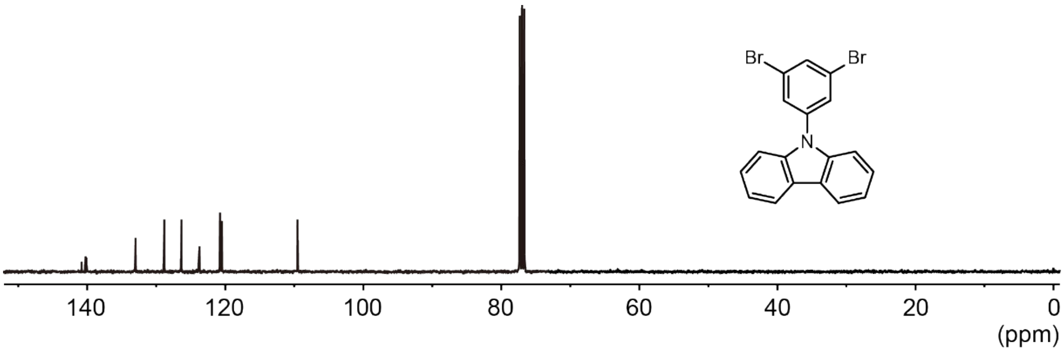

Figure S8. ^{13}C NMR spectrum of 9-(3,5-dibromophenyl)-9H-carbazole (**DBrPhCz**).

2. A brief survey of published organic afterglow molecules

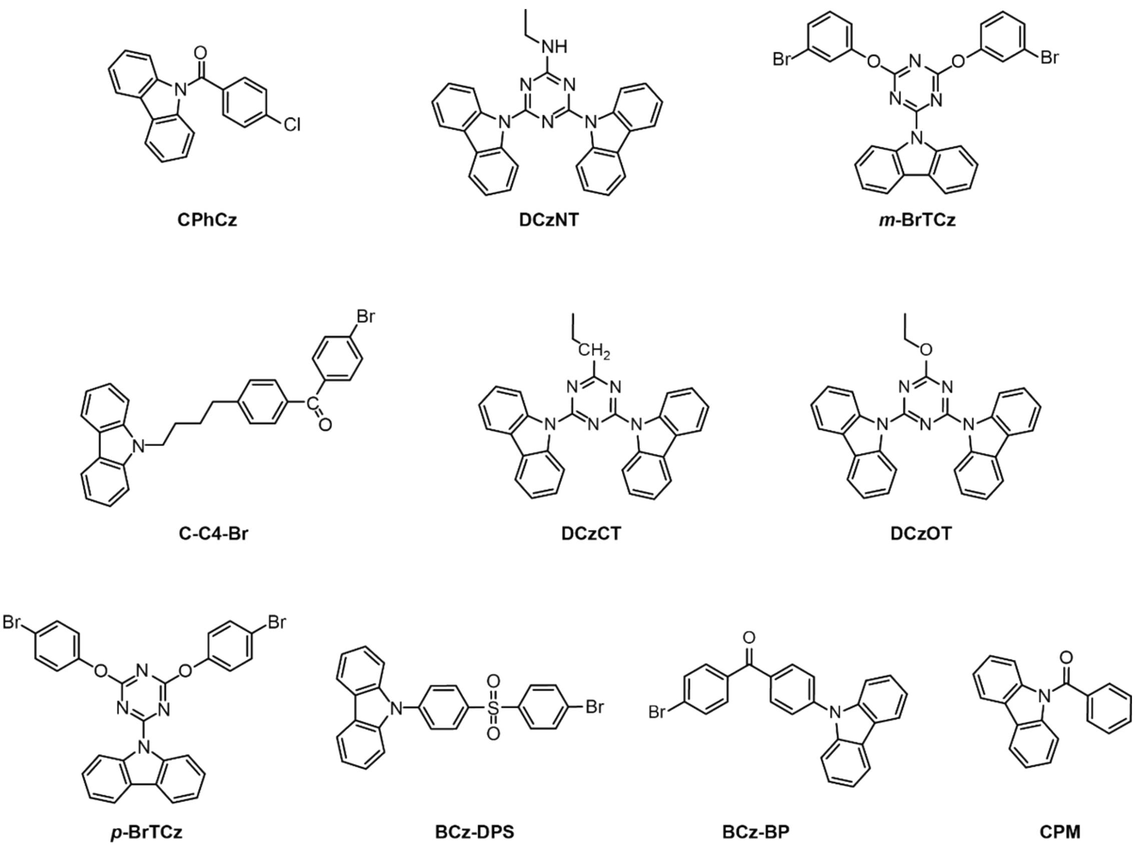

Scheme S1. Molecular structures of reported efficient organic afterglow materials of **CPhCz**[2], **DCzNT**[3], ***m*-BrTCz**[4], **C-C4-Br**[5], **DCzCT**[3], **DCzOT**[3], ***p*-BrTCz**[4], **BCz-DPS**[6], **BCz-BP**[6] and **CMP**[7] with OURTP lifetimes over 100 ms and quantum efficiencies larger than 3%.

Table S1. Excitation wavelength, OURTP lifetime (τ) and quantum efficiency (φ_p) of organic afterglow small molecules.

No.	Afterglow material	Excitation wavelength (nm)	τ (ms)	φ_p (%)	Reference
1	***p*BrPhCz**	400	250	9.5	This work
2	**CPhCz**	410	840	8.3	Ref [2]
3	***m*BrPhCz**	400	240	6.6	This work
4	**PhCz**	400	270	1.8	This work
5	**DCzNT**	365	633	15.4	Ref [3]
6	***m*-BrTCz**	365	120	13.0	Ref [4]
7	**C-C4-Br**	365	140	11	Ref [5]
8	**DCzCT**	365	651	9.7	Ref [3]
9	**DCzOT**	365	789	9.2	Ref [3]
10	***p*-BrTCz**	365	155	7.7	Ref [4]
11	**BCz-DPS**	365	120	6.0	Ref [6]
12	**BCz-BP**	365	280	5.0	Ref [6]
13	***m*BrPhCz**	295	180	3.3	This work
14	**CPM**	365	748	3.17	Ref [7]
15	***o*-BrTCz**	365	158	2.3	Ref [4]
16	**(R)/(S)-DMBDA**	360	670	2.3	Ref [8]
17	**CzDClT**	365	490	2.1	Ref [9]
18	**Cz-DPS**	365	390	2.1	Ref [6]
19	**1**	254	1850	2.0	Ref [10]
20	**CMPM**	365	340	1.98	Ref [7]
21	***p*BrPhCz**	295	200	1.5	This work
22	**PhCz**	295	240	1.5	This work
23	**CZBP**	365	518	1.40	Ref [11]
24	**DPhCzT**	365	1060	1.25	Ref [9]
25	**PhCz**	376	646	0.7	Ref [2]
26	**DECzT**	365	1350	0.6	Ref [9]
27	**CMOPM**	365	114	0.51	Ref [7]
28	**DEOPh**	254	710	0.3	Ref [9]
29	**Cz-BP**	365	490	0.3	Ref [6]
30	**DCzPhP**	365	290	0.08	Ref [9]

3. Single crystal X-ray analysis

Single crystals of ***p*BrPhCz**, ***m*BrPhCz** and **DBrPhCz** were grown from a mixture of DCM and hexane. X-ray diffraction data of these single crystals were collected on a Bruker Smart Apex CCD area detector diffractometer using graphite-monochromated

Mo-Kα radiation (λ = 0.71073 Å) at 100 K. A narrow-frame method with scan widths of 0.30° in angular velocity (ω) was applied during the data collection. Cell parameters were retrieved using SMART software and refined using SAINT on all observed reflections. Structures were solved by direct methods using the program SHELX-97 program package. Non-hydrogen atoms were found using alternating difference Fourier syntheses and least-squared refinement cycles and, during the final cycles, were refined anisotropically. Hydrogen atoms were placed in calculated positions and refined as riding atoms with a uniform value of U_{iso}. Thus obtained crystallographic parameters of ***p*****BrPhCz**, ***m*****BrPhCz** and **DBrPhCz** were summarized in Table S1 and their CCDC reference numbers are 1573611, 1573612, and 1573614, respectively.[12]

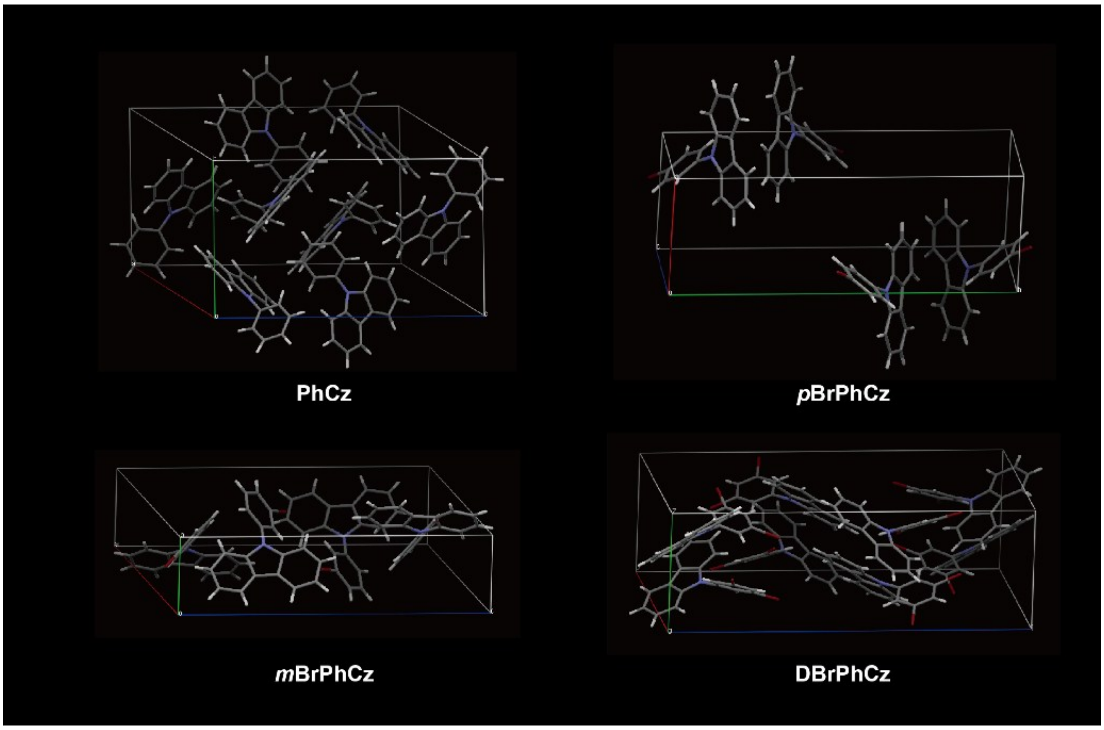

Figure S9. Single-crystal unit cells of **PhCz,** ***p*****BrPhCz,** ***m*****BrPhCz** and **DBrPhCz**.

Table S2. Crystallographic data of **PhCz**, ***p*BrPhCz**, ***m*BrPhCz**, and **DBrPhCz**.

Compound	**PhCz**[13]	***p*BrPhCz**	***m*BrPhCz**	**DBrPhCz**
Empirical formula	$C_{18}H_{13}N$	$C_{18}H_{12}NBr$	$C_{18}H_{12}NBr$	$C_{18}H_{11}NBr_2$
Formula weight (g mol^{-1})	243.1	321.0	321.0	401.1
Crystal color	colorless	colorless	colorless	colorless
Wavelength (Å)	-	0.71073	0.71073	0.71073
Space Group	P 2_1/n	P 2_1/c	P 2_1/c	P bca
a (Å)	14.526	8.4137(7)	14.1421(15)	14.9529(14)
b (Å)	10.985	20.0893(18)	5.0957(5)	8.1730(8)
c (Å)	18.094	8.6391(8)	19.509(2)	24.260(2)
α (deg)	90	90	90	90
β (deg)	113.45	108.609(2)	100.673(2)	90
γ (deg)	90	90	90	90
V (Å^3)	2648.76	1436.12(19)	1381.5(2)	2964.9(5)
Z	8	4	4	8
Density (g cm^{-3})	0.733	1.490	1.549	1.797
μ (mm^{-1})	-	2.851	2.963	5.459
T_{min}, T_{max}	-	0.726, 0.764	0.667, 0.701	0.984, 0.986
F(000)	-	648.0	1029	1564.0
h_{max}, k_{max}, l_{max}	-	11, 27, 11	18, 6, 26	19, 10, 20
$Theta_{max}$	-	28.510	28.380	28.270

4. Thermophysical and electrochemical property measurements

Differential thermal analysis (DTA) and thermogravimetric analysis (TGA) were conducted on a PERKIN-ELMER Diamond TG/DTA under a heating rate of 10ºC/min and a nitrogen flow rate of 100 mL/min.

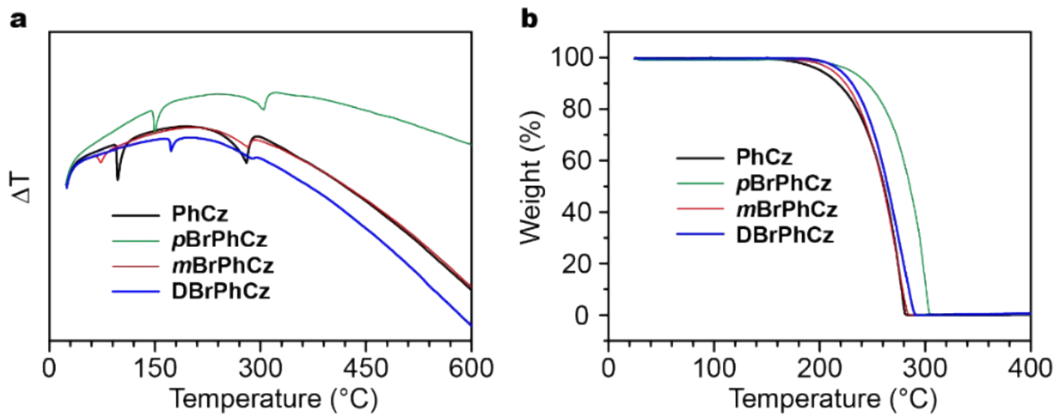

Figure S10. DTA (a) and TGA (b) curves of **PhCz**, ***p*BrPhCz**, ***m*BrPhCz**, and **DBrPhCz**.

Cyclic voltammetry (CV) measurements were carried out on a CHI660E system in a typical three-electrode cell with a working electrode (glass carbon), a reference electrode (Ag/Ag^{+}), and a counter electrode (Pt wire) in an acetonitrile solution of Bu4NPF6 (0.1 M) at a sweeping rate of 100 mV s^{-1} at room temperature.[14] The highest occupied molecular orbital (HOMO) energy level (E_{HOMO}) of the material deposited on the surface of the glass carbon working electrode can be estimated from the onset potential of the electrochemical oxidation wave based on the reference energy level of ferrocene/ferrocenium (-4.8 eV), according to Equations S1:

$$E_{HOMO} = -[E_{onset}^{Ox} - E_{(Fc/Fc^{+})} + 4.8]\, eV \qquad \text{(S1)}$$

where $E_{(Fc/Fc^{+})}$ is the onset oxidative voltage of ferrocene *vs* Ag/Ag^{+} and *EOx onset* is the onset potential of the oxidation electrochemical wave. The lowest unoccupied molecular orbital (LUMO) energy level (E_{LUMO}) can be identified by combining the E_{HOMO} and optical bandgap (E_g) in Equation S2:

$$E_{LUMO} = E_{HOMO} + E_g \qquad \text{(S2)}$$

Table S3. Thermophysical and electrochemical properties of **PhCz, *p*BrPhCz, *m*BrPhCz** and **DBrPhCz**.

**Comp.**	T_m (°C)[a]	T_d (°C)[b]	HOMO (eV)	LUMO (eV)	${}^{opt}E_g$ (eV)
**PhCz**	97	199	-5.64	-2.04	3.60
***p*BrPhCz**	150	211	-5.63	-1.99	3.64
***m*BrPhCz**	73	229	-5.62	-2.01	3.61
**DBrPhCz**	172	219	-5.62	-1.91	3.71

[a]: T_m is the melting temperature; [b]: T_d is the thermal decomposition temperature.

5. Photophysical property investigations

Ultraviolet-visible (UV-Vis) spectra were obtained using a SHIMADZU UV-3600 UV-VIS-NIR spectrophotometer. Steady-state and time-resolved photoluminescence and excitation spectra, photoluminescence lifetime decay curves, and emission quantum yields were measured on an Edinburgh FLSP920 fluorescence spectrophotometer. A xenon arc lamp (Xe900) was used as the excitation source for the steady-state photoluminescence and excitation spectra, photophysical kinetic and

quantum yields measurements. The temperature of the samples was controlled by Optistat DN2 and the emission quantum yields were measured by an integrating sphere. The phosphorescence spectra were obtained at 77 K with a 10 ms delay time after the excitation of a microsecond flash-lamp (uF900). The uF900 flash lamp produces short, typically a few μs, and high irradiance optical pulses for phosphorescence measurements in the range from microseconds to 10 seconds. Therefore, the uF900 flash lamp was also used in the measurements of the organic ultralong room temperature phosphorescence (OURTP) for organic afterglow. Excitation-phosphorescence mapping was performed on Hitachi F-4600 with an internal 5 ms delay time under ambient conditions. The lifetimes (τ) of the luminescence were obtained by fitting the decay curve with a multi-exponential decay function of

$$I(t)=\sum_i A_i e^{-\frac{t}{\tau}} \quad \text{(S3)}$$

where A_i and τ_i represent the amplitudes and lifetimes, respectively, of the individual components for multi-exponential decay profiles.[9]

The quantum yields (η) of the emission were the absolute ones done by photon counting from the excitation source into an integration sphere with the ratio of photons emitted, as descripted in the following equation.

$$\eta=\frac{N^{em}}{N^{abs}} \quad \text{(S4)}$$

In the equation, N^{em} is the number of emitted photons and N^{abs} is the number of absorbed photons. The quantum efficiency (QE) of OURTP is determined through peak-differentation-imitating analysis from the steady-state PL spectra.

The photographs and videos were recorded by a Nikon D90 camera at room temperature.

5.1 Steady-state photophysical properties

These Br-substituted **PhCz** compounds have very similar UV-Vis absorption spectra to that of **PhCz** in dilute solutions, thin films, and crystals (Figure S11), exhibiting carbazole-dominated π→π* transition for the first strong absorption band

around 290 nm in solution and 295 nm in solid and n→π* transition for the second weak absorption band around 340 nm in solution and 345 nm in solid at room temperature. It should be noted that the π→π* transition is slighted influenced by the Br substituents, while the n→π* transition can be significantly modified, leading to the blue-shifted absorption bands after Br substitution due to the electron-withdrawing effects of Br (Table S4). As to the photoluminescence spectra, **PhCz**, ***p*BrPhCz**, ***m*BrPhCz** and **DBrPhCz** show characteristic fluorescent emission bands of carbazole around 350 and 360 nm in dilute CH_2Cl_2 solution (Figures 2a-b). In solid films, the two fluorescence peaks converge into one slightly red-shifted peak around 367 nm and the excimer emission (~530 nm) can be observed in ***m*BrPhCz** and **DBrPhCz** films (Figures 2c-d), owing to their dense molecular packing after multiple Br substitution.

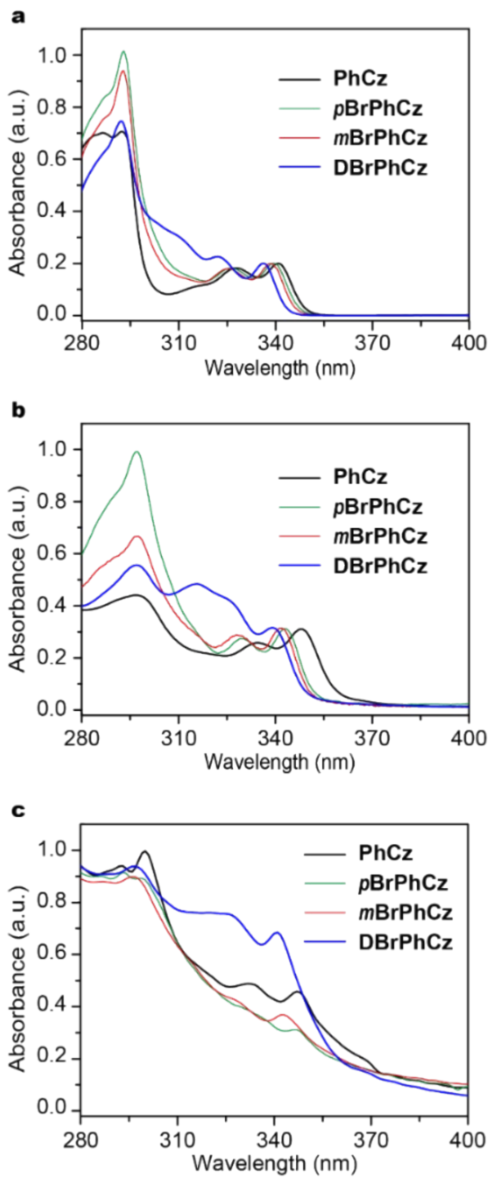

Figure S11. UV-vis absorption spectra of **PhCz**, ***p*BrPhCz**, ***m*BrPhCz** and **DBrPhCz** in CH_2Cl_2 dilute (~10^{-5} mol L^{-1}) solution (a), thin film (b), and crystal (c) at room temperature.

Table S4. Absorption peaks of **PhCz**, ***p*BrPhCz**, ***m*BrPhCz** and **DBrPhCz** in CH_2Cl_2 solution, thin film and crystal.

Compd.	Solution	Film	Crystal
	λ_{abs} (nm)	λ_{abs} (nm)	λ_{abs} (nm)
**PhCz**	292, 327, 341	298, 335, 348	300, 332, 347
***p*BrPhCz**	293, 326, 339	297, 329, 343	297, 327, 342
***m*BrPhCz**	293, 325, 338	297, 329, 343	299, 330, 347
**DBrPhCz**	292, 322, 335	297, 315, 339	297, 325, 341

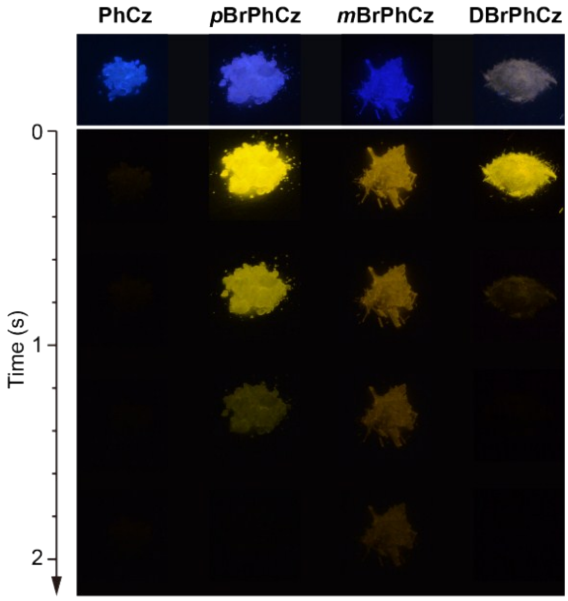

Figure S12. Photographs of **PhCz,** ***p*****BrPhCz,** ***m*****BrPhCz** and **DBrPhCz** powders taken at different time intervals before (first row) and after (succeeding rows) turn-off of the photoexcitation (365 nm) under ambient conditions.

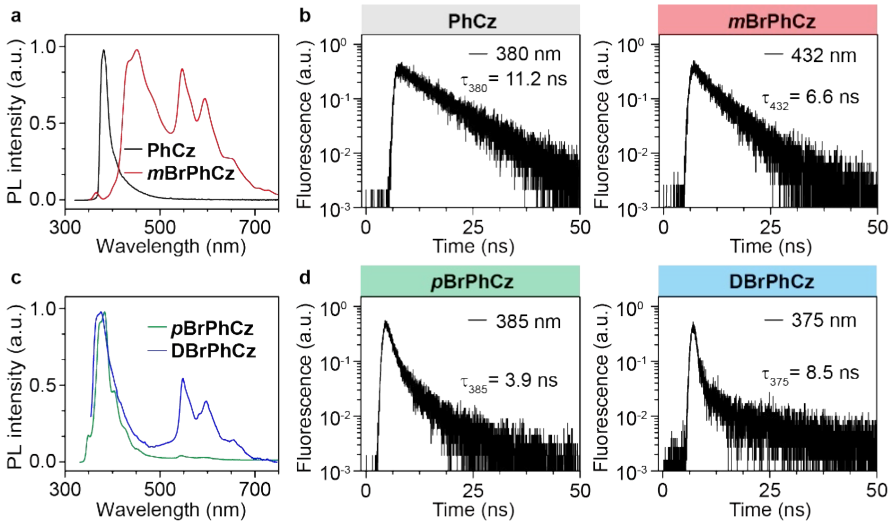

Figure S13. Steady-state emission spectra (a, c) and the time-resolved fluorescence decay curves (b, d) of **PhCz,** ***m*****BrPhCz**, ***p*****BrPhCz,** and **DBrPhCz** crystals with excitation wavelength of 295 nm at room temperature.

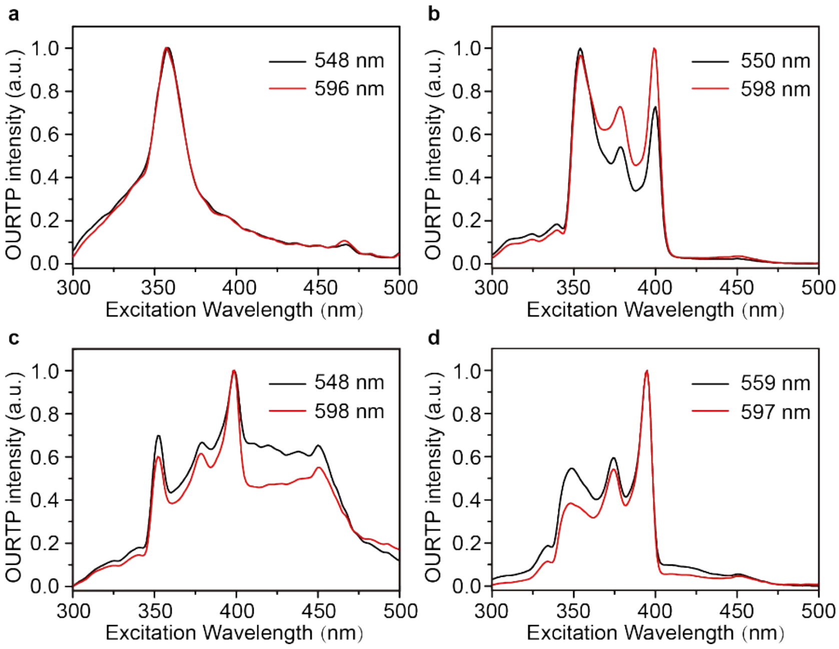

Figure S14. Excitation Spectra of **PhCz** (a), ***p*BrPhCz** (b), ***m*BrPhCz** (c) and **DBrPhCz** (d) crystals for their OURTP peaks at 77 K.

5.2 Time-resolved photoluminescence properties

The OURTP emission excited by either 295 or 400 nm is very stable in both strength and lifetime at different atmospheres of nitrogen, air and oxygen (Figure S17). However, the 400 nm excitation needs more time (~0.5 s) to populate T_1 for steady OURTP emissions than 295 nm excitation (0.1 s), indicating also that different photophysical procedures occur under the two different excitation wavelength (Figure S18): the one-step $S_0 \rightarrow T_1$ absorption to populate T_1 through visible-light excitation is slower than the traditional multi-step UV-light excited process, including $S_0 \rightarrow S_n$ absorption, internal conversion (IC), and ISC. Therefore, when the excitation intensity increases, the steady-state and afterglow emissions by 295 nm excitation can be strengthened linearly, but they will be saturated at high intensities of 400 nm excitation (Figure S19); also, due to weak spin-forbidden $S_0 \rightarrow T_1$ absorption upon visible light excitation, the 400 nm excited OURTP emission needs more time (1.0 s) to reach constant intensity than that by 295 nm excitation (0.1 s), according to the excitation duration tests (Figure S20).

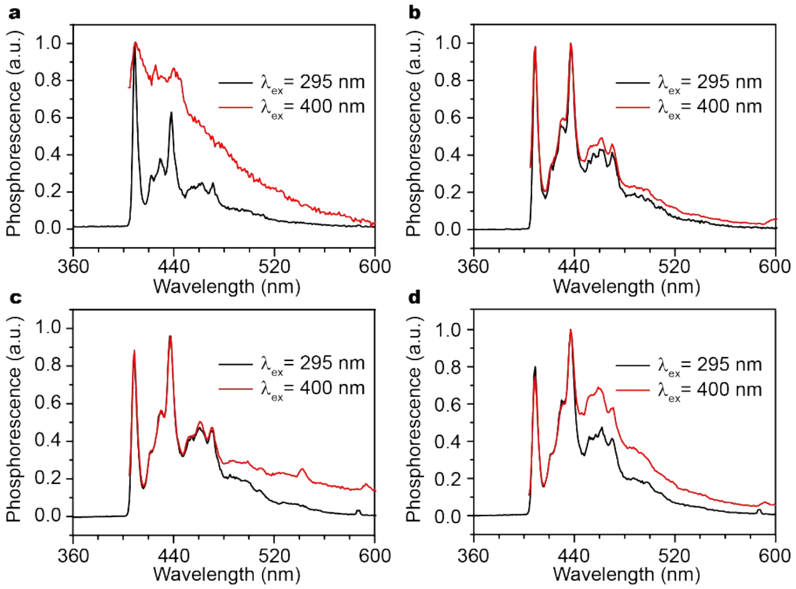

Figure S15. UV-light (295 nm, black line) and visible-light (400 nm, red line) excited phosphorescence spectra of **PhCz** (a)**,** ***p*BrPhCz** (b)**,** ***m*BrPhCz** (c) and **DBrPhCz** (d) in 2-methyltetrahydrofuran dilute (~10^{-5} mol L^{-1}) solution at 77 K with a delay time of 10 ms.

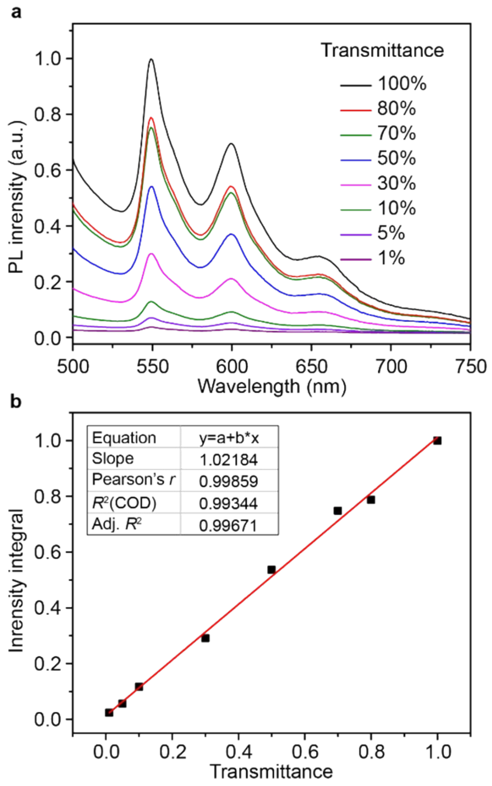

Figure S16. (a) PL spectra at varied excitation transmittance of ***p*BrPhCz** crystal at 400 nm; (b) A close linear dependence of intensity integral of OURTP emission band with excitation transmittance.

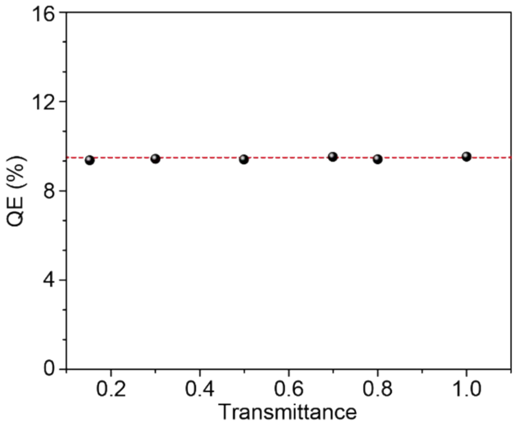

Figure S17. OURTP QEs of ***p*BrPhCz** crystal under different transmittance of 400 nm excitation under ambient conditions.

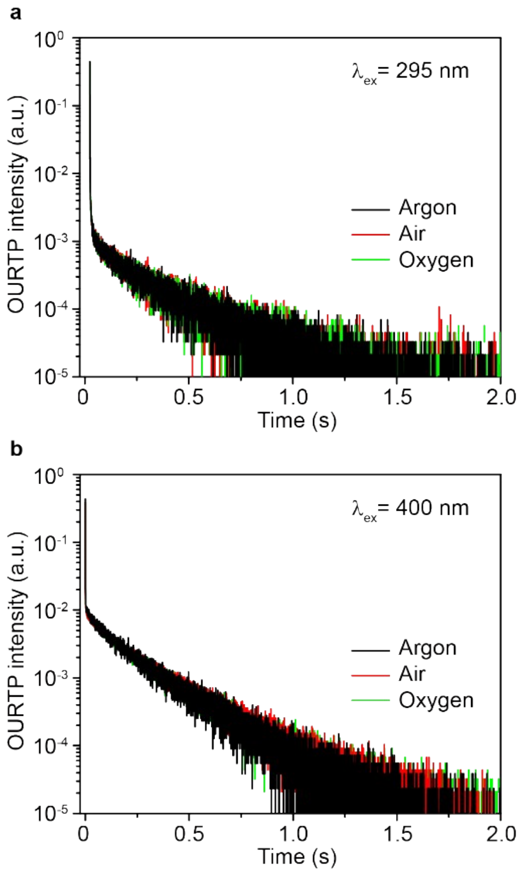

Figure S18. Time-resolved OURTP (550 nm) decay curves of ***p*BrPhCz** crystal excited by 295 (a) and 400 nm (b) light in different atmosphere (argon, air, and oxygen) under ambient conditions.

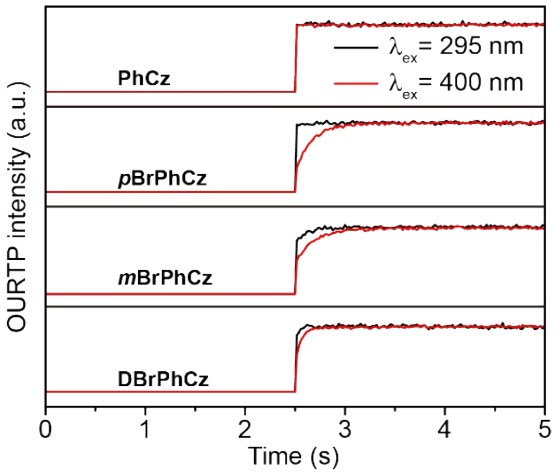

Figure S19. Time-dependent intensity profiles of the OURTP emissions of **PhCz** (548 nm), ***p*BrPhCz** (550 nm), ***m*BrPhCz** (548 nm) and **DBrPhCz** (549 nm) crystals excited by 295 and 400 nm light under ambient conditions. The excitation is turned on at 2.5 s and maintained for 2.5 s at room temperature.

5.3 Excitation strength and duration consideration

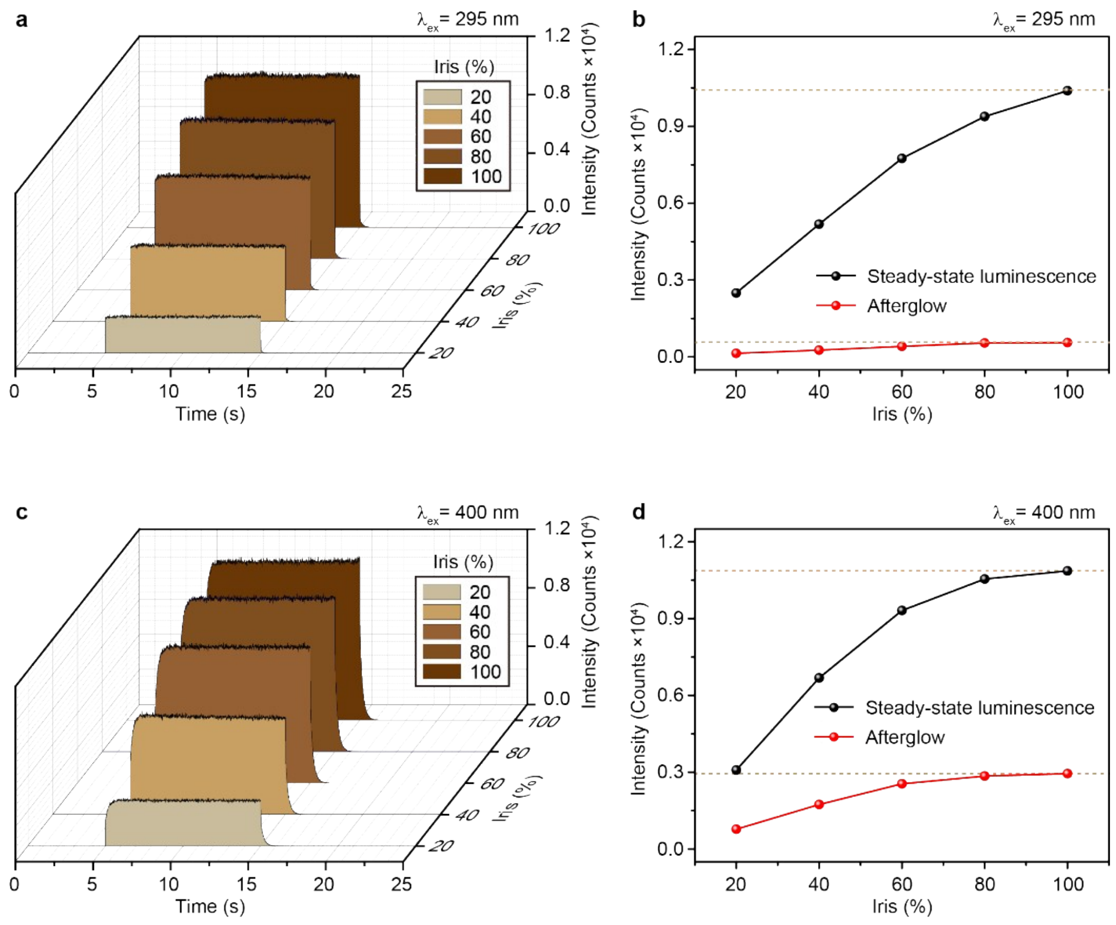

Figure S20. The influence of excitation strength on OURTP emission (550 nm) of *p*BrPhCz crystal under ambient conditions. Intensity profile of the 550 nm emission (a, c) as a function of time and different Iris (20, 40, 60, 80 and 100 %) and the strength of the steady-state and afterglow OURTP emission (b, d) excited at 295 (a, b) and 400 nm (c, d) at room temperature. The sample was irradiated for 10 s, during which time the steady state luminescence (550 nm) strength was measured. The OURTP intensity was measured by collecting all the afterglow emission photons when the excitation source was switched off.

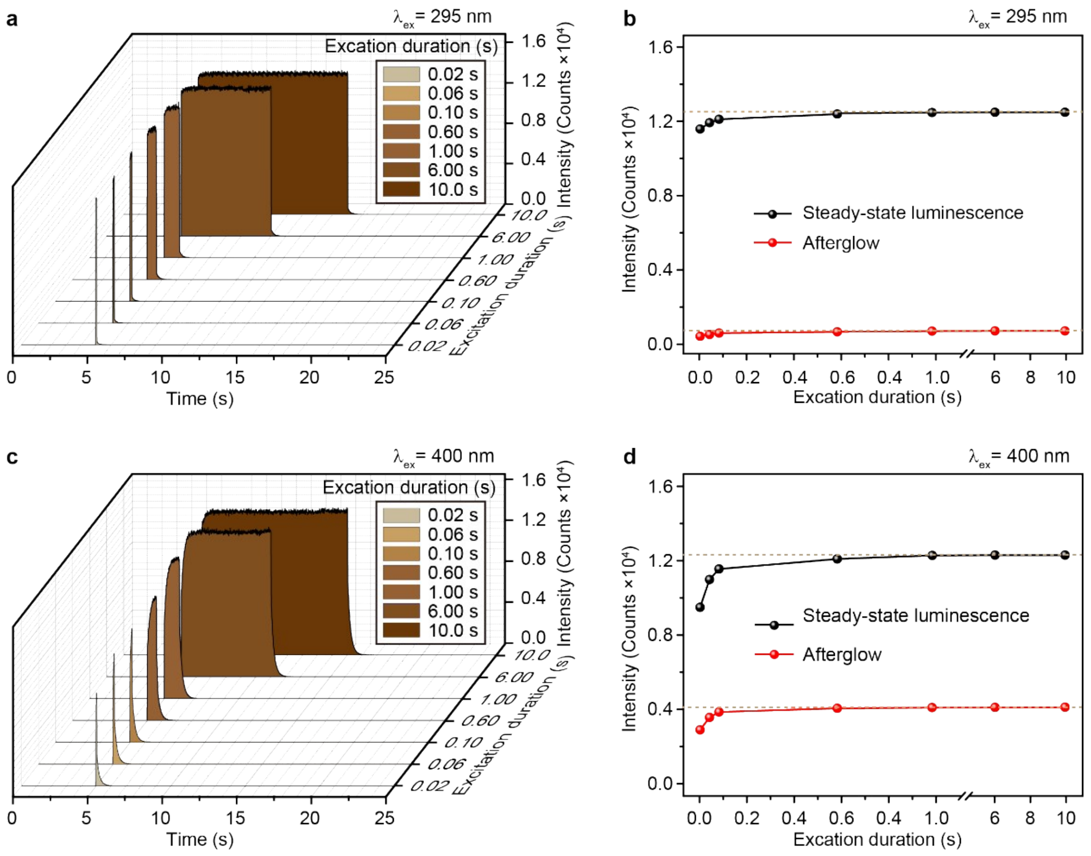

Figure S21. The influence of excitation duration on OURTP emission (550 nm) of *p*BrPhCz crystal under ambient conditions. Intensity profiles of the 550 nm emission as a function of time (a, c) and the corresponding intensities of the steady-state and afterglow OURTP emission (b, d) upon excitation of 295 (a, b) and 400 nm (c, d) light with different irradiating time under ambient conditions. The sample was irradiated for 0.02, 0.06, 0.1, 0.6, 1.0, 6.0 and 10 s, respectively.

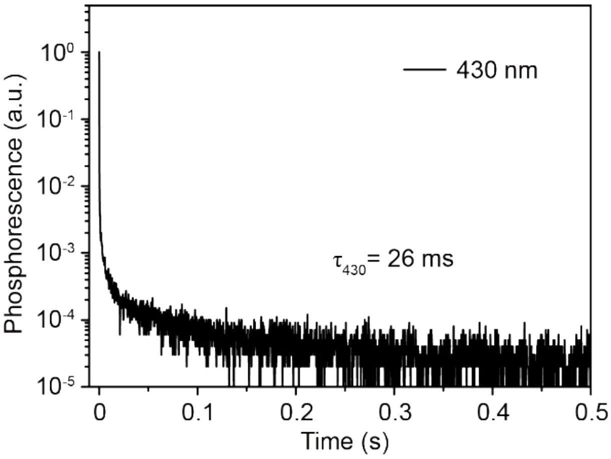

Figure S22. Time-resolved phosphorescence (430 nm) decay curve of the ***p*BrPhCz** crystal with excitation wavelength of 400 nm at room temperature.

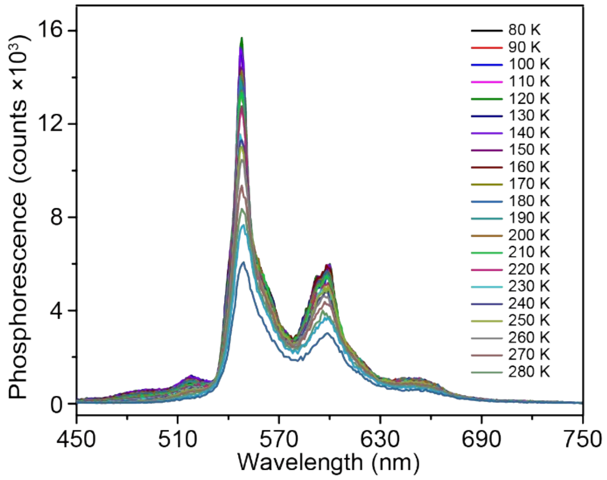

Figure S23. Temperature dependence phosphorescence curves of the ***p*BrPhCz** crystal under excitation wavelength of 400 nm with a delay time of 10 ms.

The lifetime of an excited state is determined by the relaxation rates of all its decay channels, but if the lifetime of its population source is longer, the lifetime of the luminophor will be dominated by the population source.[15] When the lifetime of T_n* is longer than that of the intrinsic lifetime of T_1* at 77 K, longer organic afterglow emission lifetime is resulted under 295 nm excitation at 77 K, since the long-lived T_n* participates the population process of T_1* for afterglow emission and dominates the afterglow lifetime under 295 nm excitation (Table S5). The intrinsic lifetime of T_1* can be measured under 400 nm excitation to exclude the influence of T_n* (Table S5). At the

room temperature, the lifetime of T_n* is significantly decreased, leading to close lifetime under 295 or 400 nm excitation; the lightly shorter lifetime under 295 nm excitation could be due to the enhanced non-radiative decay rates of T_1*, since the organic afterglow compounds upon 295 nm excitation would result in larger molecular geometry changes for looser molecular packing than that under 400 nm excitation with small geometry variation.[16]

Table S5. Emission peaks (λ) and lifetimes (τ) of the OURTP emission after excitation of 295 and 400 nm light at 77 K.

Compd.	**λ_{ex}=295 nm**				**λ_{ex}=400 nm**			
	λ (nm)	τ (s)	λ (nm)	τ (s)	λ (nm)	τ (s)	λ (nm)	τ (s)
**PhCz**	548	1.16	596	1.07	548	0.59	596	0.46
***p*BrPhCz**	550	0.54	598	0.52	550	0.40	598	0.42
***m*BrPhCz**	548	0.45	598	0.41	548	0.41	598	0.42
**DBrPhCz**	549	0.22	597	0.22	549	0.08	597	0.07

6. Mechanism study

6.1 Probing the formation of triplet excited states

The existence of triplet states in the solution of Br-substituted **PhCz** derivatives was detected by photodegradation of anthracene-9,10-diyl-bis-methylmalonate (ADMA). In principle, the energy transfer between the triplet excited state of the compound and the ground state of molecular oxygen ($^3O_2$) will lead to the generation of an electronically excited state of molecular oxygen, *i.e.* singlet oxygen ($^1O_2$), upon photoexcitation. Therefore, ADMA can be used to detect the generated $^1O_2$ as a chemical trap and this trapping process can be quantitatively monitored using a UV-vis spectrophotometer. Specifically, if there are triplet states and $^1O_2$ generated in the system, the characteristic absorption peaks of ADMA (359, 378, and 399 nm) will gradually decrease in intensity and eventually disappear.[17]

To confirm the generation of triplet excited states upon 400 nm excitation, the experiments were conducted on well dissolved **PhCz and DBrPhCz** molecules (20 μM) in tetrahydrogenfuran (THF) containing ADMA (50 μM) and dissolved molecular oxygen. A control experiment (Figure S23) with only ADMA in THF (50 μM) was also performed and there is a negligible change in the absorption spectra of ADMA after excitation at 400 nm for 45 min.

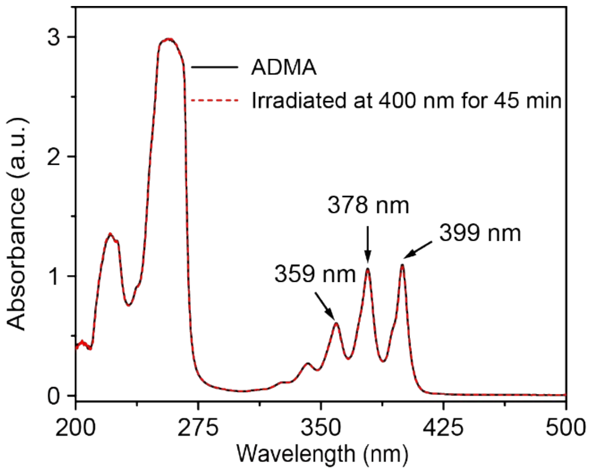

Figure S24. Absorption spectra of ADMA (50 μM) in THF under irradiation at 400 nm for 45 min at room temperature.

6.2 Control experiment

Control experiments were also performed on a previously reported afterglow molecule of **DNCzP** (Figure S24a-b) to check the unique $S_0 \rightarrow T_1$ absorption in these heavy atom and heteroatom incorporated molecules. **DNCzP** has a similar phosphorescent spectrum (Figure S24c) to ***p*BrPhCz**, but it cannot be excited by the visible light (λ_{ex}>355 nm) for either phosphorescent emission (Figure S24d).

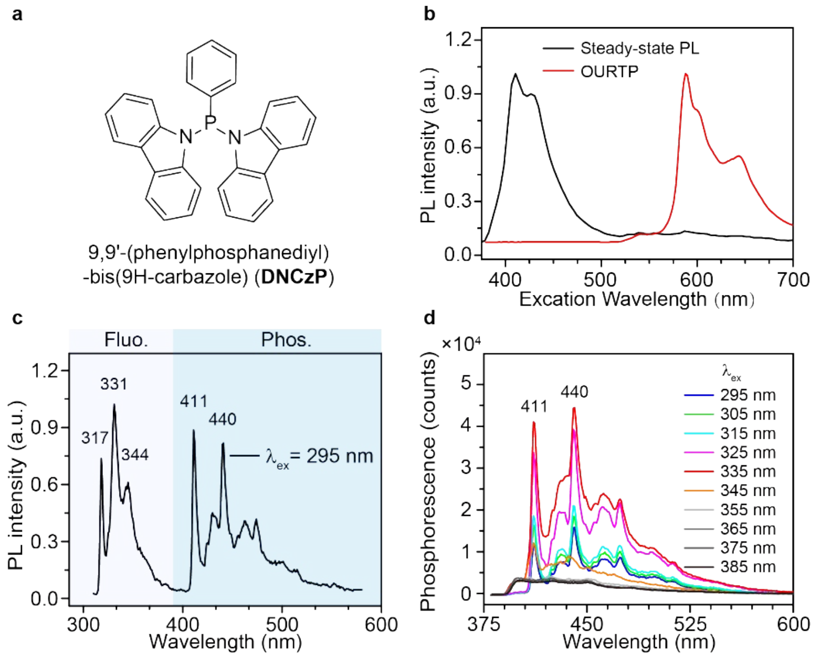

Figure S25. Molecular structure of **DNCzP** (a), photoluminescence of the **DNCzP** on excitation of 365 nm in crystal at room temperature (b), photoluminescence of the **DNCzP** on excitation of 295 nm (c) and phosphorescence spectra excited from 295 to 375 nm in 2-methyltetrahydrofuran dilute solution (~10^{-5} mol L^{-1}) at 77 K with a delay time of 10 ms (d).

6.3 Theoretical calculations

Density functional theory (DFT) and time-dependent DFT (TD-DFT) calculations were carried out at the B3LYP/6-31G(d) level using on Gaussian 09 package.[18] The ground state (S_0) geometry was fully optimized with B3LYP/6-31G(d) and the optimized stationary point was further characterized by harmonic vibration frequency analysis to ensure that real local minima had been found. The excitation energies in the *n*-th singlet (S_n) and *n*-th triplet (T_n) states were obtained using the TD-DFT method based on an optimized molecular structure at ground state (S_0). Spin-orbit coupling (SOC) matrix elements between the singlet and triplet excited states are calculated with quadratic response function methods using the Dalton program at the optimized geometry of the lowest singlet excited state (S_1) using B3LYP functional and 6-31G(d) basis set.[19, 20] The SOC between the ground state and the lowest triplet excited state (T_1)

was calculated using the same method but was based on the optimized ground state geometry.

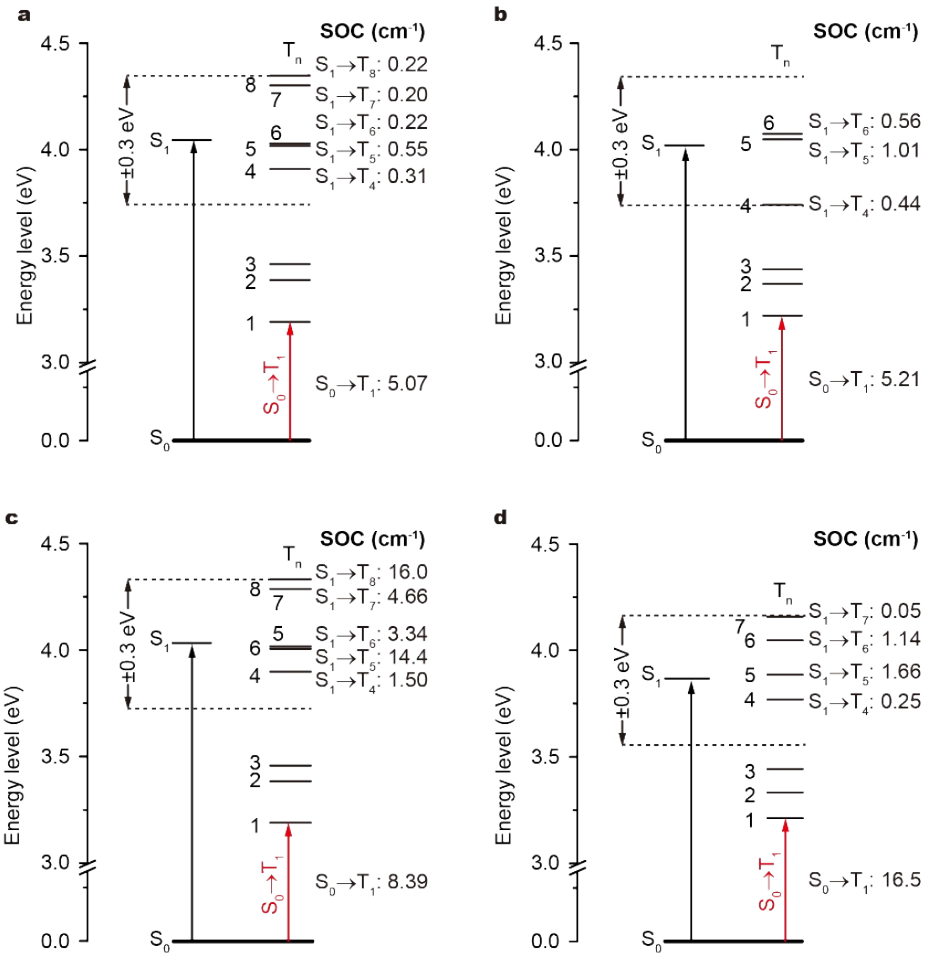

Figure S26. Schematic diagrams showing the TD-DFT-calculated energy levels at singlet (S_n) and triplet (T_n) states and spin-orbit coupling (SOC) values for $S_1 \rightarrow T_n$ and $S_0 \rightarrow T_n$ of **PhCz** (a), ***p*BrPhCz** (b), ***m*BrPhCz** (c) and **DBrPhCz** (d)**.** Note that only the most possible T_n with singlet-triplet splitting (ΔE_{ST}) lower than 0.3 eV were illustrated.

The intermolecular interactions between the heavy atom of Br and the π-conjugated moiety of the nearby molecule were investigated by nonbonding covalent interaction (NCI)[21] analysis using Multiwfn version 3.3[22] software based on typical molecular packing structures in single crystals with B3LYP/6-31G(d). NCI isosurface plots were performed with color scaling that the dark blue color represents an attractive

interaction, while dark red color represents repulsive interactions. The plotted isosurfaces were demonstrated with reduced density gradient (RDG) of 0.5 and -0.05 < sign(λ_2)ρ < 0.05, where sign(λ_2) means the sign of the second largest eigenvalue of Hessian and ρ represents the electron density.

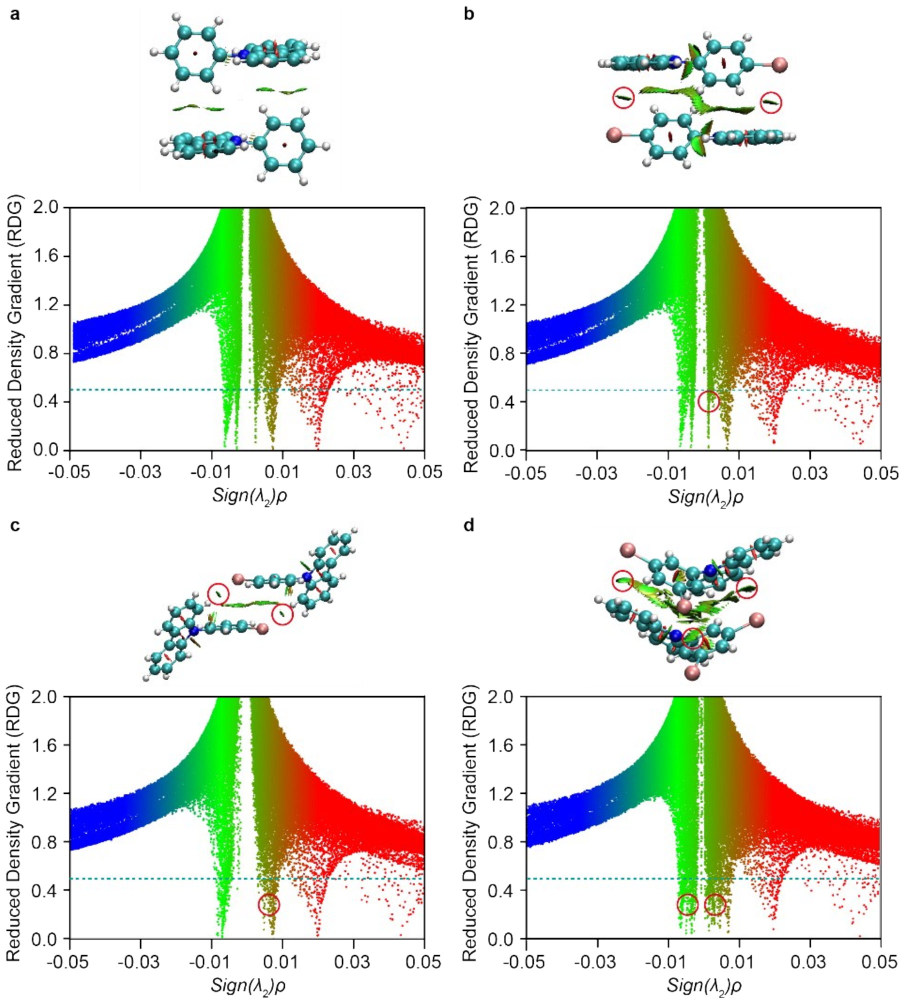

Figure S27. Plots of reduced density gradient (RDG) versus sign(λ_2)ρ with RDG isosurface of the selected dimers in **PhCz** (a), ***p*BrPhCz** (b), ***m*BrPhCz** (c) and **DBrPhCz** (d) single crystals. Intermolecular heavy atom interactions were highlighted in red cycles.

6.4 Photophysical modeling

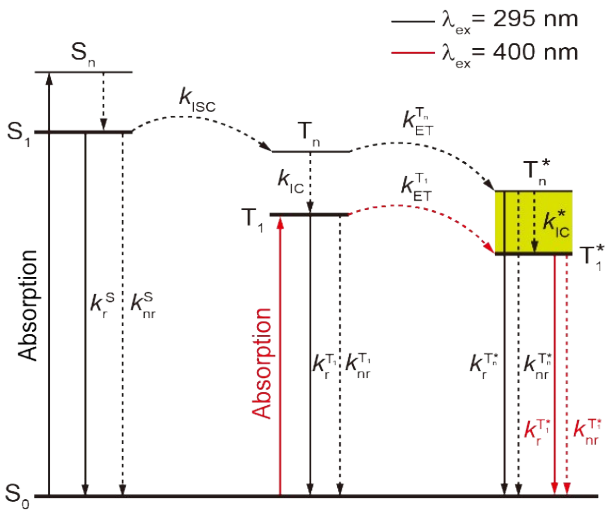

Figure S28. Photophysical processes for the organic afterglow excited by UV-light at 295 nm or visible-light at 400 nm. Note that *k*S r and *k*S nr are the radiative and non-radiative decay rate constants of S_1 respectively; k_{ISC} is the ISC rate constant; kT_1 r and kT_1 nr are the radiative and non-radiative decay rate constants of T_1; $kT_1{}^*$ rand $kT_1{}^*$ nr are the radiative and non-radiative decay rate constants of stabilized T_1 (T* 1); kT_1 ET and *k*Tn ET are the energy trapping rate constants of T_1 and T_n; $kT_n{}^*$ rand $kT_n{}^*$ nr are the radiative and non-radiative decay constants of stabilized T_n (T* n); k_{IC} and *k** IC are the rate constants of internal conversion rate constants of T_n and T* n respectively.

Theoretically, the concentration decay of S_1, T_n, T_1, T* n, and T* 1 after the removal of the UV-excitation source at 295 nm can be described in Equations S5-9 under consideration of their all decay channels as illustrated in Figure S28.

$$\frac{d[S_1]}{d[t]} = -\left(k_r^S + k_{nr}^S + k_{ISC}\right)[S_1] \tag{S5}$$

$$\frac{d[T_n]}{d[t]} = k_{ISC}[S_1] - \left(k_{ET}^{T_n} + k_{IC}\right)[T_n] \tag{S6}$$

$$\frac{d[T_1]}{d[t]} = k_{IC}[T_n] - \left(k_r^{T_1} + k_{nr}^{T_1} + k_{ET}^{T_1}\right)[T_1] \tag{S7}$$

$$\frac{d[T_n^*]}{d[t]} = k_{ET}^{T_n}[T_n] - \left(k_r^{T_n^*} + k_{nr}^{T_n^*} + k_{IC}^*\right)[T_n^*] \tag{S8}$$

$$\frac{d[T_1^*]}{d[t]} = k_{IC}^*[T_n^*] + k_{ET}^{T_1}[T_1] - \left(k_r^{T_1^*} + k_{nr}^{T_1^*}\right)[T_1^*] \tag{S9}$$

The solutions of these differential equations can be expressed in biexponential forms as

$$[\mathrm{S_1}] = \mathrm{C_1} \exp\left({}^{-t}\!/_{\tau_1} \right) \tag{S10}$$

$$[\mathrm{T_n}] = \mathrm{C_2} \exp\left({}^{-t}\!/_{\tau_2} \right) + \mathrm{C_3} \exp\left({}^{-t}\!/_{\tau_1} \right) \tag{S11}$$

$$[\mathrm{T_1}] = \mathrm{C_4} \exp\left({}^{-t}\!/_{\tau_3} \right) + \mathrm{C_5} \exp\left({}^{-t}\!/_{\tau_2} \right) + \mathrm{C_6} \exp\left({}^{-t}\!/_{\tau_1} \right) \tag{S12}$$

$$[\mathrm{T_n^*}] = \mathrm{C_7} \exp\left({}^{-t}\!/_{\tau_4} \right) + \mathrm{C_8} \exp\left({}^{-t}\!/_{\tau_2} \right) + \mathrm{C_9} \exp\left({}^{-t}\!/_{\tau_1} \right) \tag{S13}$$

$$[\mathrm{T_1^*}] = \mathrm{C_{10}} \exp\left({}^{-t}\!/_{\tau_1} \right) + \mathrm{C_{11}} \exp\left({}^{-t}\!/_{\tau_2} \right) + \mathrm{C_{12}} \exp\left({}^{-t}\!/_{\tau_3} \right) + \mathrm{C_{13}} \exp\left({}^{-t}\!/_{\tau_4} \right) + \mathrm{C_{14}} \exp\left({}^{-t}\!/_{\tau_5} \right) \tag{S14}$$

where,

$$\tau_1 = \frac{1}{k_{\mathrm{r}}^{S} + k_{\mathrm{nr}}^{S} + k_{\mathrm{ISC}}} \tag{S15}$$

$$\tau_2 = \frac{1}{k_{\mathrm{IC}} + k_{\mathrm{ET}}^{\mathrm{T_n}}} \tag{S16}$$

$$\tau_3 = \frac{1}{k_{\mathrm{r}}^{\mathrm{T_1}} + k_{\mathrm{nr}}^{\mathrm{T_1}} + k_{\mathrm{ET}}^{\mathrm{T_1}}} \tag{S17}$$

$$\tau_4 = \frac{1}{k_{\mathrm{r}}^{\mathrm{T_n^*}} + k_{\mathrm{nr}}^{\mathrm{T_n^*}} + k_{\mathrm{IC}}^{*}} \tag{S18}$$

$$\tau_5 = \frac{1}{k_{\mathrm{r}}^{\mathrm{T_1^*}} + k_{\mathrm{nr}}^{\mathrm{T_1^*}}} \tag{S19}$$

Under visible light (400 nm) excitation, the concentration decay of T_1 and T* 1 after removing the excitation source can be written in Equations S20-21 (Figure S28).

$$\frac{\mathrm{d[T_1]}}{\mathrm{d[t]}} = -\left(k_{\mathrm{r}}^{\mathrm{T_1}} + k_{\mathrm{nr}}^{\mathrm{T_1}} + k_{\mathrm{ET}}^{\mathrm{T_1}}\right)[\mathrm{T_1}] \tag{S20}$$

$$\frac{\mathrm{d[T_1^*]}}{\mathrm{d[t]}} = k_{\mathrm{ET}}^{\mathrm{T_1}}[\mathrm{T_1}] - \left(k_{\mathrm{nr}}^{\mathrm{T_1^*}} + k_{\mathrm{r}}^{\mathrm{T_1^*}}\right)[\mathrm{T_1^*}] \tag{S21}$$

The solutions of these differential equations are as follows in biexponential forms

$$[\mathrm{T_1}] = \mathrm{C_{15}} \exp\left({}^{-\mathrm{t}}\!/_{\tau_6} \right) \tag{S22}$$

$$[\mathrm{T_1^*}] = \mathrm{C_{16}} \exp\left({}^{-\mathrm{t}}\!/_{\tau_7} \right) + \mathrm{C_{17}} \exp\left({}^{-\mathrm{t}}\!/_{\tau_6} \right) \tag{S23}$$

where,

$$\tau_6 = \frac{1}{k_{\mathrm{r}}^{\mathrm{T_1}} + k_{\mathrm{nr}}^{\mathrm{T_1}} + k_{\mathrm{ET}}^{\mathrm{T_1}}} \tag{S24}$$

$$\tau_7 = \frac{1}{k_r^{T_1^*} + k_{nr}^{T_1^*}} \tag{S25}$$

According to this photophysical model, multi-lifetimes should be observed from the luminescence decay curves of phosphorescence and OURTP. However, in most cases, shorter lifetime cannot be fitted from the experimental decay curves, which should be due to the large difference between these lifetimes and the larger one generally dominates the luminescence decay measured by time-correlated single photon counting (TCSPC) technology using a microsecond flash-lamp.

7. Aggregation structure analysis

Aggregation structures influence significantly on the photophysical properties of organic optoelectronic materials. Especially, H-aggregation plays an important role in stabilizing the triplet excitons to elongate their lifetime for the realization of organic afterglow at room temperature. To probe the existence of H-aggregation, the aggregation structure analysis was performance on the single crystal structures of these OURTP molecules. According to the molecular exciton theory, the exciton splitting energy ($\Delta\varepsilon$) in dimer is given by[9]:

$$\Delta\varepsilon = \frac{2|M|^2}{r_{uv}^3}(\cos\alpha - 3\cos\theta_1\cos\theta_2) \tag{S26}$$

where M is the electric dipole transition moment, α is the angle between the transition moments of the two molecules in the dimer, and θ_1 and θ_2 are the angles between transition moments of the two molecules and the interconnection of the centers respectively, r_{uv} is the distance between the molecular pair. And, when $\Delta\varepsilon > 0$, it belongs to H-aggregation, and when $\Delta\varepsilon < 0$, it is J-aggregation.

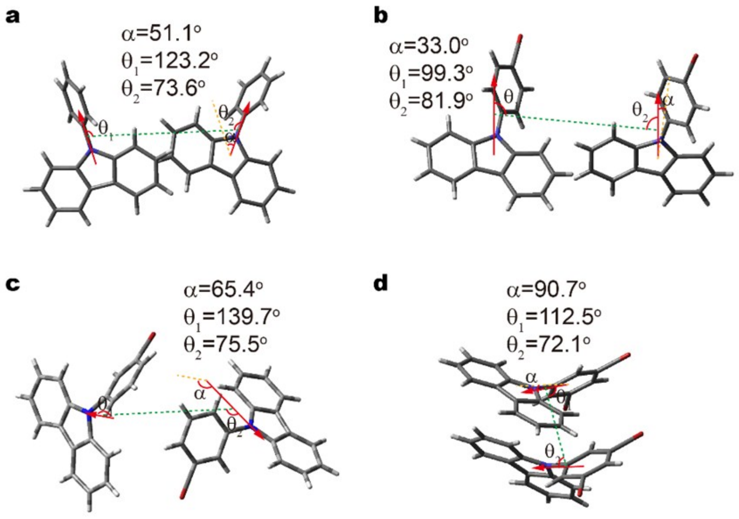

Figure S29. Typical H-aggregated structures of **PhCz** (a), ***p*BrPhCz** (b), ***m*BrPhCz** (c), and **DBrPhCz** (d) dimers in their single crystal structures.

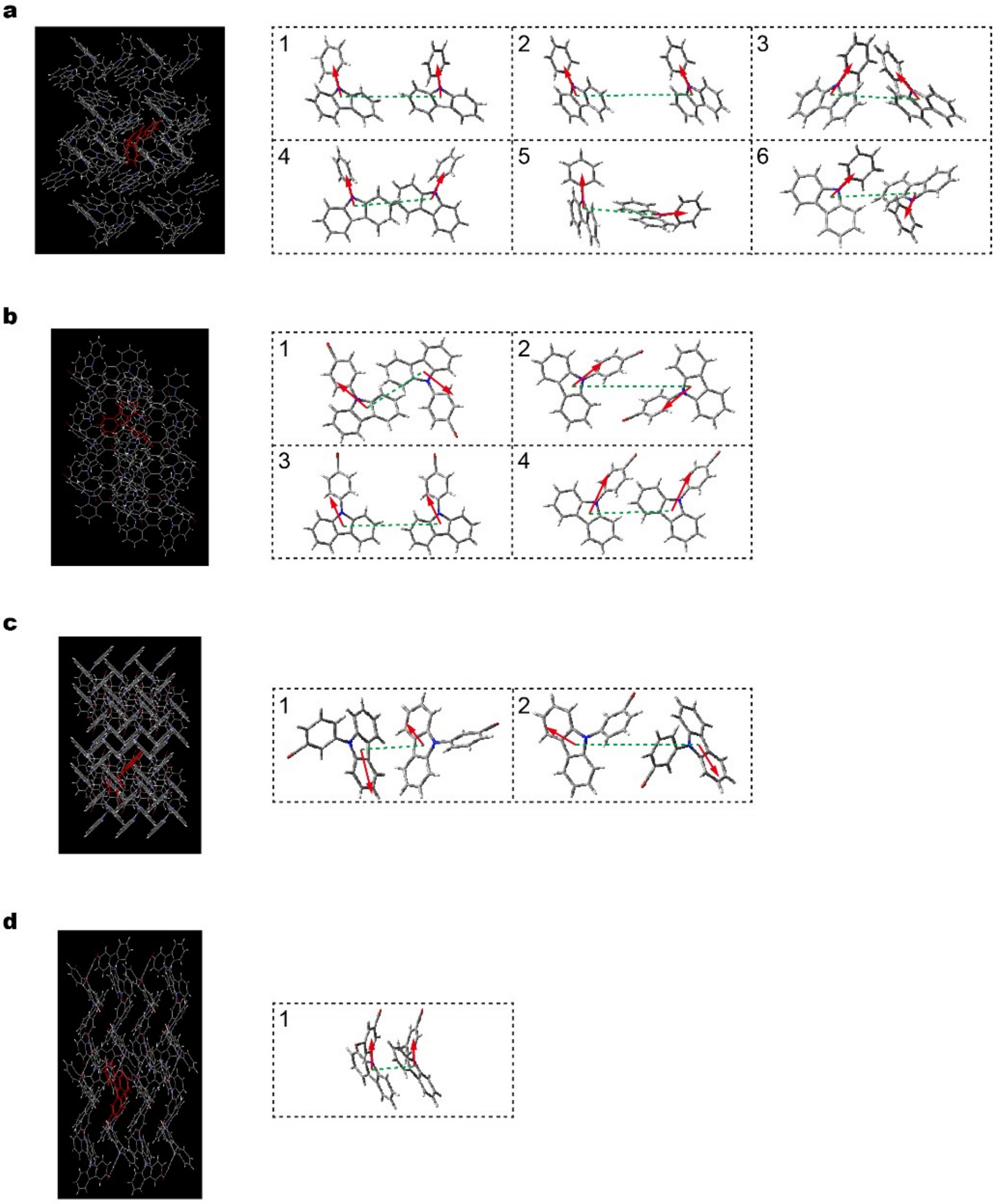

Figure S30. Various H-aggregates in **PhCz** (a), ***p*BrPhCz** (b), ***m*BrPhCz** (c), and **DBrPhCz** (d) crystals selected by positive exciton splitting energy (Δε) according to the molecular exciton theory.

Table S6. H-aggregates (H, in red) and J-aggregates (J, in black) in **PhCz**, ***p*BrPhCz**, ***m*BrPhCz**, and **DBrPhCz** crystals identified by the positive and negative exciton splitting energy (Δε) respectively.

Compd.	Aggregation	α (°)	θ_1 (°)	θ_2 (°)	M (Debye)	r_{uv} (Å)	$\Delta\varepsilon$ (eV)
**PhCz**	H-1	50.9	106.9	91.1	3.61	10.16	0.0094
	H-2	0.0	64.5	64.5	3.61	10.98	0.0056
	H-3	85.4	66.7	118.8	3.61	8.49	0.0175
	H-4	51.1	123.2	73.6	3.61	9.63	0.0200
	H-5	94.6	150.7	79.8	3.61	8.52	0.0100
	H-6	128.9	66.5	128.4	3.61	9.41	0.0025
	J-1	180.0	83.2	96.8	3.61	9.21	-0.0200
	J-2	129.1	127.8	94.4	3.61	9.21	-0.0163
	J-3	85.4	37.3	48.1	3.61	10.70	-0.0200
	J-4	51.1	22.5	51.1	3.61	8.91	-0.0256
	J-5	94.6	102.5	160.9	3.61	9.18	-0.0150
***p*BrPhCz**	H-1	33.0	99.3	81.9	5.13	10.19	0.0281
	H-2	180.0	131.3	48.7	5.13	12.19	0.0056
	H-3	180.0	54.4	125.6	5.13	12.11	0.0006
	H-4	0.0	66.8	66.8	5.13	10.06	0.0175
	J-1	180.0	71.9	108.1	5.13	8.75	-0.0350
	J-2	0.0	20.5	20.5	5.13	8.66	-0.0831
***m*BrPhCz**	H-1	65.40	81.4	135.4	10.92	9.86	0.1144
	H-2	65.40	139.7	75.5	10.92	10.46	0.1288
	J-1	114.6	82.9	118.5	10.92	10.98	-0.0269
	J-2	0.0	147.3	147.3	10.92	5.10	-1.2650
	J-3	65.4	107.5	157.8	10.92	12.21	-0.0344
	J-4	65.4	165.0	101.4	10.92	12.70	-0.0113
**DBrPhCz**	H-1	90.7	112.5	72.1	3.58	6.66	0.0188
	J-1	89.1	86.6	3.6	3.58	11.12	-0.0019
	J-2	4.9	134.5	134.6	3.58	12.41	-0.0038
	J3	175.1	97.3	84.9	3.58	11.58	-0.0100
	J4	89.3	99.5	171.2	3.58	10.69	-0.0063

8. Flexible pattern encryption application

Time-resolved and color-encoded encrypted images were fabricated as follows. On a flexible polyethylene terephthalate (PET) substrate, a thin film of a blue short-lived thermally activated delayed fluorescence (TADF) emitter of bis[4-(9,9-dimethyl-9,10-dihydroacridine)phenyl]sulfone (DMAC-DPS)[23] dispersed in ALOE VERA gel (c.a. 40 mg/mL) was deposited firstly by silk-screen printing. In the second step, the pattern of 'NJUPT' logo was printed on the surface of DMAC-DPS film by the same technology using the red fluorescent molecule of 10-(2-benzothiazolyl)-2,3,6,7-tetrahydro-1,1,7,7-tetramethyl-1H,5H,11H-(1)benzopyropyrano(6,7-8-I,j)quinolizin-11-one (C545T)[24] dispersed also in ALOE VERA gel (c.a. 40 mg/mL). Then, the pattern encryption device was turned over and on the other side of PET substrate, the organic afterglow molecule of ***p*BrPhCz** in ALOE VERA gel was silk-screen printed to paint the encrypted pattern of the 'IAM' logo. On photoexcitation, the 'NJUPT' logo in red fluorescence of C545T appears on the blue light emitting background of DMAC-DPS; the interference of blue fluorescence of ***p*BrPhCz** was successfully eliminated by the DMAC-DPS layer. When the excitation source is turned off, the encrypted pattern of 'IAM' logo can be clearly observed by naked eyes under ambient conditions at room temperature (see Supporting Video).

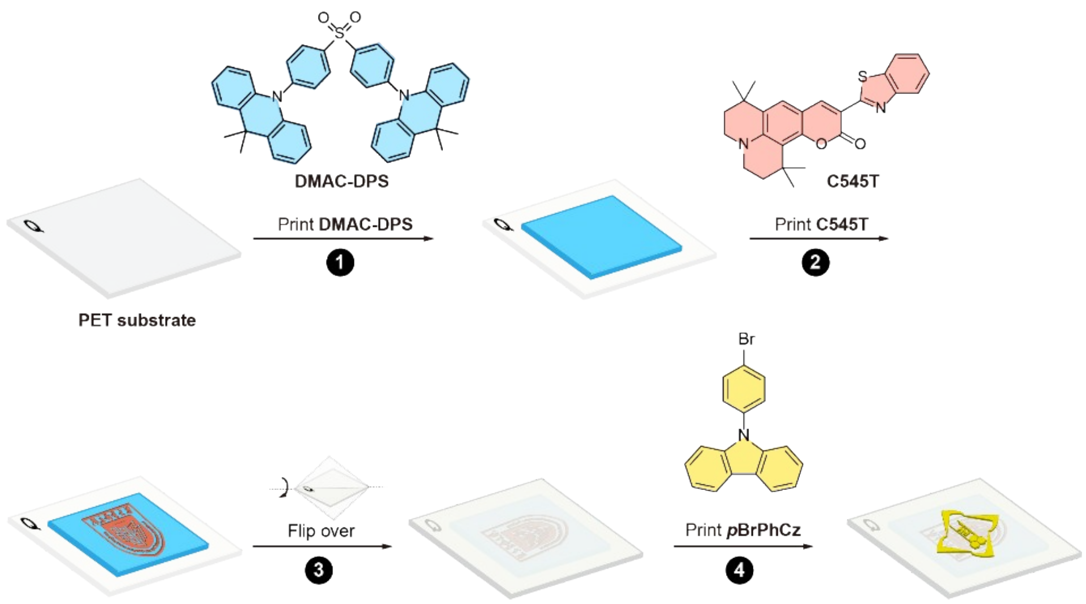

Figure S31. Fabrication of the time-resolved and color-encoded encrypted images on flexible substrate in four steps. Firstly, a thin layer of blue TADF emitter of DMAC-DPS was printed on the front surface of PET flexible substrate. Secondly, 'NJUPT' log was printed using red fluorescent dye of C545T on the surface of DMAC-DPS film. Thirdly, the pattern encryption device was turned over. Finally, the 'IAM' logo was printed on the back surface of the PET substrate using the organic afterglow molecule of ***p*BrPhCz** dispersed in ALOE VERA.

Reference:

1. J. Hassan, M. Sevignon, C. Gozzi, E. Schulz and M. Lemaire, *Chem. Rev.*, 2002, **102**, 1359-1470.
2. S. Cai, H. Shi, J. Li, L. Gu, Y. Ni, Z. Cheng, S. Wang, W. w. Xiong, L. Li, Z. An and W. Huang, *Adv. Mater.*, 2017, **29**, 1701244-1701250.
3. L. Gu, H. Shi, C. Miao, Q. Wu, Z. Cheng, S. Cai, M. Gu, C. Ma, W. Yao, Y. Gao, Z. An and W. Huang, *J. Mater. Chem. C*, 2018, **6**, 226-233.
4. Y. Tao, J. J. Xiao, C. Zheng, Z. Zhang, M. K. Yan, R. F. Chen, X. H. Zhou, H. H. Li, Z. F. An, Z. X. Wang, H. Xu and W. Huang, *Angew. Chem. Int. Ed.,* 2013, **52**, 10491-10495.
5. S. M. A. Fateminia, Z. Mao, S. Xu, Z. Yang, Z. Chi and B. Liu, *Angew. Chem. Int. Ed.*, 2017, **56**, 12160-12164.
6. Z. Yang, Z. Mao, X. Zhang, D. Ou, Y. Mu, Y. Zhang, C. Zhao, S. Liu, Z. Chi, J. Xu, Y. C. Wu, P. Y. Lu, A. Lien and M. R. Bryce, *Angew. Chem. Int. Ed.*, 2016, **55**, 2181-2185.
7. Y. Xie, Y. Ge, Q. Peng, C. Li, Q. Li and Z. Li, *Adv. Mater.*, 2017, **29**, 1606829-606836.
8. S. Hirata and M. Vacha, *J. Phys. Chem. Lett.*, 2016, **7**, 1539-1545.
9. Z. An, C. Zheng, Y. Tao, R. Chen, H. Shi, T. Chen, Z. Wang, H. Li, R. Deng, X. Liu and W. Huang, *Nature mat.,* 2015, **14**, 685-693.
10. Y. Shoji, Y. Ikabata, Q. Wang, D. Nemoto, A. Sakamoto, N. Tanaka, J. Seino, H. Nakai and T. Fukushima, *J. Am. Chem. Soc.*, 2017, **139**, 2728-2733.
11. Y. Gong, G. Chen, Q. Peng, W. Z. Yuan, Y. Xie, S. Li, Y. Zhang and B. Z. Tang, *Adv. Mater.*, 2015, **27**, 6195-6201.
12. Z. Sun, J. Luo, S. Zhang, C. Ji, L. Zhou, S. Li, F. Deng and M. Hong., *Adv. Mater.* 2013, **25**, 4159-4163.
13. C. Avendaño, M. Espada, B. Ocaña, S. García-Granda, M. D. R. Díaz, B. Tejerina, F. Gómez-Beltrán, A. Martínez and J. Elguero, *J. Chem. Soc., Perkin Trans*, 1993, **8**, 1547-1555.

14. Y. Tao, L. Xu, Z. Zhang, R. Chen, H. Li, H. Xu, C. Zheng and W. Huang, *J. Am. Chem. Soc.,* 2016, 138, 9655-9662.
15. X. Liu, Y. Wang, X. Li, Z. Yi, R. Deng, L. Liang, X. Xie, D. T. B. Loong, S. Song, D. Fan, A. H. All, H. Zhang, L. Huang and X. Liu, *Nature Comm.*, 2017, **8**, 899-905.
16. Y. S. Park, K. H. Kim and J. J. Kim, *Appl. Phys. Lett.*, 2013, **102**, 153306-153310.
17. W. Hu, M. Xie, H. Zhao, Y. Tang, S. Yao, T. He, C. Ye, Q. Wang, X. Lu and W. Huang, *Chem. Sci.*, 2017, **9**, 999-1005.
18. M. J. Frisch, G. W. Trucks, H. B. Schlegel, G. E. Scuseria, M. A. Robb, J. R. Cheeseman, J. A. Montgomery, T. Jr Vreven, K. N. Kudin, J. C. Burant, Gaussian 09 Gaussian Inc: Wallingford, CT, **2009**.
19. Dalton, a molecular electronic structure program. Dalton: 2011, available online: http://daltonprogramorg
20. Q. Peng, Q. Shi, Y. Niu, Y. Yi, S. Sun, W. Li and Z. Shuai, *J. Mater. Chem. C*, 2016, **4**, 6829-6838.
21. Y. Cheng, Y. Qi, Y. Tang, C. Zheng, Y. Wan, W. Huang and R. Chen, *J. Phys. Chem. Lett.*, 2016, **7**, 3609-3615.
22. T. Lu and F. Chen, *J. Comput. Chem.*, 2012, **33**, 580-592.
23. Q. Zhang, B. Li, S. Huang, H. Nomura, H. Tanaka and C. Adachi, *Nature Photo.*, 2014, **8**, 326-332.
24. X. Liu, Z. Chen, C. Zheng, M. Chen, W. Liu, X. Zhang and C. S. Lee, *Adv. Mater.*, 2015, **27**, 2025-2030.
